# Prediction of Pellet Durability Index in a commercial feed mill using multiple linear regression with variable selection and dimensionality reduction

**DOI:** 10.1093/jas/skaf021

**Published:** 2025-02-04

**Authors:** Jihao You, Dan Tulpan, Cheryl Krziyzek, Jennifer L Ellis

**Affiliations:** Department of Animal Biosciences, University of Guelph, Guelph, ON, Canada; Department of Animal Biosciences, University of Guelph, Guelph, ON, Canada; Trouw Nutrition Canada, Guelph, ON, Canada; Department of Animal Biosciences, University of Guelph, Guelph, ON, Canada

**Keywords:** Forward Selection, Partial Least Squares, Pellet Durability Index, prediction model, Principal Component Analysis

## Abstract

Pellet quality, measured as the Pellet Durability Index (**PDI**), is an important key performance indicator for commercial feed manufacturing, as it can impact both mill efficiency and downstream performance of animals fed the manufactured diets. However, it is an ongoing challenge for the feed industry to control pellet quality, due to the complexity of feed manufacturing and the large number of variables influencing the process. Previous studies have explored the prediction of pellet quality using either simple empirical models with a few variables or machine learning models with many variables. The objective of the current study was to develop statistical regression models to predict PDI and to describe the relationship between pellet quality and 55 available variables based on a dataset with 2691 observations collected from a commercial feed mill. In the current study, the response variable (PDI) was transformed using the Box–Cox approach into the transformed response variable (tPDI), which was more normally distributed. Three multiple regression models were developed based on subsets of variables processed by variable selection and dimensionality reduction methods: Forward Selection, Principal Component Analysis, and Partial Least Squares. The results indicated that Model 1 (Forward Selection with manual removal of sparse variables), built on 9 variables, performed better than the other 2 models. It exhibited consistent model prediction performance on the training data and testing data, in terms of mean absolute error (1.93 ± 0.063 vs. 1.96), RMSPE (2.45 ± 0.079 vs. 2.45), and concordance correlation coefficient (0.549 ± 0.0273 vs. 0.550), with a better prediction precision based on the fit plot. *Expanding Temperature (°C)*, *Fat Content (%)*, *ADF Content (%)*, and *Indoor Humidity (Pelletizer) (%*) were identified as more influential than other variables on the transformed response variable (tPDI) in Model 1, based on a behavior analysis. The models developed in the current study can be helpful to feed mills for predicting and comprehending the effect of a number of commonly measured variables on pellet quality in the commercial setting.

## Teaser

This study explored the relationship between pellet durability index and a large number of commercially available variables using statistical modeling approaches. The models developed in this study can be helpful for commercial feed mills in predicting and comprehending the effect of various aspects of formulation and manufacturing on pellet quality.

## Introduction

Pelleted feeds, produced by compacting and extruding ingredients or mixtures through die openings by pressure, heat, and moisture (Okewole and Igbeka, 2016), offer animals a homogeneous diet without ingredient segregation compared with mash feed. Animals consuming pelleted feeds perform better than those consuming mash feed, due to improved nutrient digestibility, feed consumption, and palatability (Cavalcanti and Behnke, 2005a). However, pelleted feeds can break down during transportation and handling, undermining their potential to enhance animal performance and increasing waste via the production of fines (Myers et al., 2013). There have been numerous studies published that focus on the individual variables that impact pellet quality, such as ingredient selection, particle size, conditioning temperature, and more (Briggs et al., 1999; Gilpin et al., 2002; Buchanan and Moritz, 2009; Buchanan et al., 2010; Rasyid et al., 2019; Tillman et al., 2020). For example, Gilpin et al. (2002) reported the effect of different levels of moisture (12% and 14%), retention time (short and long), steam quality (70%, 80%, 90%, and 100%), as well as their 2-way and 3 way interactions, on pellet quality. Tillman et al. (2020) investigated the effect of changing levels of variables like conditioning temperature, inclusion of DDGS, inclusion of meat and bone meal, etc., on pellet quality. However, most studies have used 1-way or 2-way analysis of variance (**ANOVA**) to investigate a more limited number of variables (generally studies may examine between 1 and 4 treatments, with levels of the treatments also considered). In these studies, specific values of individual treatments are set at predefined levels and the effect of these on the pellet quality of the feed is examined. The objective of such studies is generally to determine the optimal level of treatments that would result in the desired or improved pellet quality. However, it is questionable whether these findings can be directly applied to the commercial feed mill setting, as such studies are generally conducted under controlled experimental settings, and only a few factors change at specific levels in each study while other factors are not examined.

Determining pellet quality at the commercial feed mill before shipping is a common practice across the feed manufacturing industry to assess and predict the durability and integrity of pelleted feeds before dispersal (Muramatsu et al., 2015). Pellet Durability Index (**PDI**) is a commonly used measure to represent pellet quality and represents the percentage of the weight of the remaining pellets after a durability test (e.g., tumbling or air blowing) to the initial weight of pellets (ASAE, 1997). This methodology is also applied experimentally, for example in the studies mentioned above (Gilpin et al., 2002; Tillman et al., 2020) to measure pellet quality. The PDI measure is designed to be a metric of what may happen to the pellets subsequently during handling and transportation, in order to get more immediate feedback on quality issues while the feed is still being manufactured. From the perspective of the feed industry, models capturing the relationship between pellet quality (PDI) and a number of these manufacturing/formulation variables known to impact PDI could provide practical benefits such as improving pellet quality and optimizing the manufacturing process preemptively or in real time.

To date, and to our knowledge, there have been two published studies that have built prediction models based on the relationship between PDI and various mill and diet factors. The study conducted by Schroeder et al. (2020) was a pioneering study in exploring the variables (number of variables = 50) affecting PDI, as empirical regression models were constructed not only to predict the pellet quality but also to identify influential factors in an industrial pelleting process. Following this study, a study from our research group (You et al., 2022) recently reported using machine learning regression models to predict PDI based on a dataset (number of variables = 16) collected from a commercial feed mill.

The objective of the current study was to construct multiple regression models using a statistical modeling approach for predicting the PDI for a commercial feed mill. Unlike previous studies using manually collected data, an automated data collection system was applied in the feed mill to record data for each pellet run, including manufacturing parameters (**MP**) and feed ingredient inclusion levels. The automatically collected data, along with data regarding nutrient composition (**NC**) of feedstuffs and environmental variables (indoor temperature/humidity), were used for modeling PDI. Compared with previous studies, the current study used a larger dataset including a wider range of variables (number of variables = 55) for potential modeling. It was hypothesized that there is a significant relationship between these variables and PDI, and that these variables could be used to predict PDI.

## Materials and Methods

### Data collection

The data used in this study was obtained from Trouw Nutrition Canada’s feed mill located in St. Marys, Ontario (Plant 2). Specifically, the data was obtained from Line 1 (Line 1 has a production capacity of 18 tons per hour for pelleted feed) of Plant 2 (Plant 2 is equipped with a double-helix horizontal mixer and can produce up to 850 tons of pelleted feed per day). The data collection period spanned from December 15, 2021, to December 6, 2022. The data was not available for the period from June 28, 2022, to July 5, 2022. There were 2,691 observations in the original dataset, where each observation represented a batch of pelleted feed. The raw data comprised 4 main parts:

1. Pelleted feed key performance indicator (**KPI**) report: This report was a combination of data captured from the automation, operator manual input, and calculations for each batch of pelleted feed. It included not only MP of each pellet run, such as conditioning temperature, expanding time, cooling time, and cumulative production, but also the usage of ingredients in the diet for each pellet run, such as the inclusion of corn, wheat, soybean meal, etc. The full list of recorded data available (manufacturing conditions and ingredients) is provided in Table S1.
2. Environmental data: Data loggers (EL-USB-2-LCD data loggers from Lascar Electronics, USA) were installed at 6 locations in the feed mills to record temperature and humidity hourly over the entire data collection period. In previous work by You et al. (2022), the average regional outdoor temperature was identified as important in PDI prediction by the best models. As a result, to explore this relationship further, the above-mentioned data loggers were installed in the mill. The locations for probe placement included areas around the pelletizer, around the conditioner, below the cooler, around the cooler exhaust fan, around the post-pellet liquid application (PPLA) elevator, and outdoor (outside the feed plant). The location of the data loggers is illustrated in Figure S1, and the list of recorded environmental data sources is provided in Table S1, labeled as **EF** for environmental factors. The internal EF recorded by the data loggers reflects the temperature and humidity resulting from the combination of heat and moisture emissions from the equipment, the profile of outdoor air being pulled inside the mill for cooling, as well as internal ambient air circulation.
3. Nutrient composition data: This data included NC (as fed) of the ingredients used for diet formulation, including DM, fat, crude protein, crude fiber, ash, ADF, NDF, and starch content. NC was measured using Near-Infrared Spectroscopy or wet chemistry methods and updated weekly, biweekly, or monthly over the entire data collection period (frequency was ingredient-specific). The list of nutrients measured is reported in Table S1 under the NC category.
4. The PDI was measured in the same way as reported in our previous study (You et al., 2022). Specifically, 100 g of pelleted feed was placed in the testing chamber of a Holmen NHP100 portable pellet durability tester where blown air caused the pellets to collide with each other. Given the abrasive nature of the process, pellet fines were removed from the screen during testing using air jets. After 60 s, the intact pellets that remained in the chamber were removed from the tester and manually weighed. The PDI was reported as a percentage, representing the weight of the pellets measured after tumbling to the weight of the pellets before tumbling. The PDI measurement was recorded by mill operators into the KPI report for each pelleting run.

The raw data sources underwent combination, including 2 main steps: 1) Aligning environmental data with the pelleted feed KPI report by calculating the average temperature and average humidity using the hourly environmental data across the production time of each batch of pelleted feed; and 2) Aligning the NC data with the data from step 1 by calculating the NC of each batch of pelleted feeds, based on the ingredient usage in the diet and NC of those ingredients. After combining the manufacturing, environmental and nutrition data, the processed dataset consisted of 55 variables categorized into 4 classes: MP (12 variables), feed ingredients (FI, 23 variables), NC (8 variables), EF (12 variables). Detailed descriptions of all variables can be found in Table S1.

### Data processing

**Figure 1.**
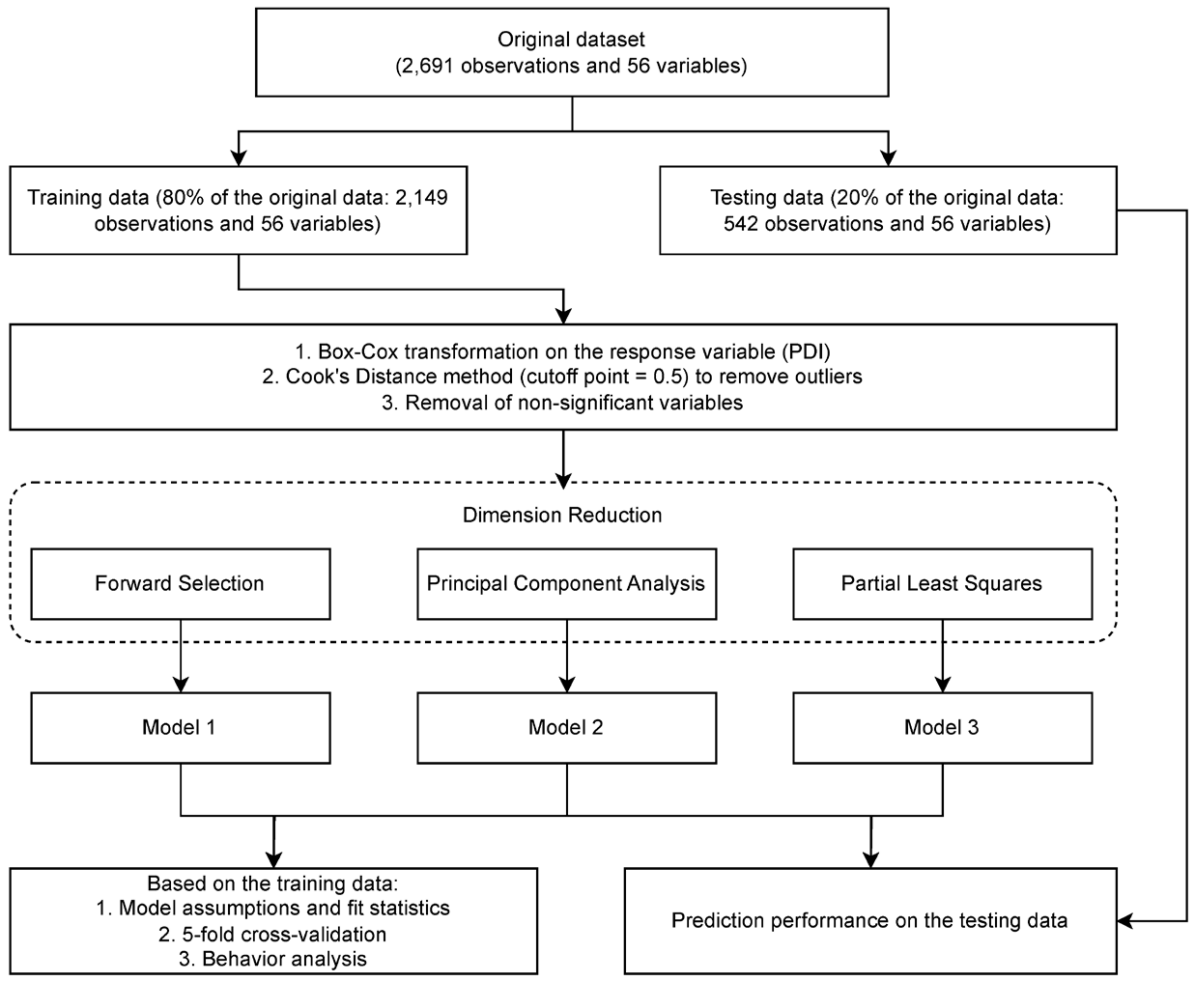
Workflow diagram of the current study.

Figure 1 shows the workflow diagram of the current study. All data analysis processes were conducted using SAS Studio version 3.81 (SAS Institute, Cary, NC).

The original dataset was randomly split into 2 parts, with 80% used for training (2,149 observations) and 20% for testing (542 observations). The model construction was based on the training data, and the model prediction was evaluated based on the testing data. In the current study, a “variable” refers to a measurement in the data collected from the feed mill; a “factor” refers to the components transformed by principal component analysis (**PCA**) or partial least squares (**PLS**); and a “predictor” refers to the variables or factors used for model construction.

Due to the skewed distribution of the response variable (PDI), the Box–Cox approach (Box and Cox, 1964) was implemented to transform the response variable (PDI) using the PROC TRANSREG procedure, which transforms the outcome variable based on the following equation:


$$\begin{array}{l} y^{\left(\lambda \right)}=\;\left\{ \begin{array}{l} \frac{y^{\lambda }-1}{\lambda }\;\left(\lambda \neq 0\right)\;\ln(y)\;\left(\lambda =0\right)\; \end{array}\right. \end{array}$$ (1)

where *y* represents the original response variable (PDI); $y^{\left(\lambda \right)}$ represents the transformed response variable (tPDI); *λ* represents the parameter that determines the power of the transformation, which was estimated using the PROC TRANSREG procedure in SAS within a range from −10 to 10 with an incremental step of 0.01. The optimal value of *λ* is determined by maximizing the log-likelihood function for the transformed data to best approximate a normal distribution.

The back-transformed predicted variable was obtained using the equation below:


$$\begin{array}{l} y_{b}=\;\left\{ \begin{array}{l} \sqrt[{\lambda }]{\lambda y^{\left(\lambda \right)}+1}\;\left(\lambda \neq 0\right)\;e^{y^{\left(\lambda \right)}}\;\left(\lambda =0\right)\; \end{array}\right. \end{array}$$ (2)

where $y_{b}$ represents the back-transformed predicted response variable (PDI); $y^{\left(\lambda \right)}$ represents the predicted (transformed scale) response variable (tPDI); and *λ* represents the parameter that determines the power of the transformation, which was estimated in the Box–Cox transformation.

The Cook’s Distance statistic (CD) was used to detect outliers during the modeling process, as it provides a measure of an observation’s effect on the fitted response values of the regression when a particular observation is excluded from the analysis (Cook, 1977). The PROC MIXED procedure was used to implement the Cook’s distance statistic test on all observations. The cutoff point for the CD statistic in this study (i.e., for an observation to be considered a potential influential outlier) was set at 0.5 (Blatná, 2006), rather than the sometimes used cutoff point of 4/*n* (where *n* is the number of observations). The 4/*n* rule is not suited to large datasets, because each data point in large datasets has a relatively smaller impact on the model. During the outlier detection process, observations with a Cook’s distance value greater than 0.5 were sequentially removed from the dataset one at a time, considering their influential impact on the model.

The significance of univariable regression slopes determined using the PROC MIXED procedure in SAS for tPDI against each individual variable was assessed, and only significant variables (*P* < 0.01) were retained for multivariable modeling.

### Variable selection and dimensionality reduction

Due to high dimensionality and potential multicollinearity in the data, 3 methods were implemented to reduce the number of variables considered for model building, including forward selection, PCA, and PLS:

1. The Forward Selection method was implemented using the PROC GLMSELECT module of SAS (Rodriguez, 2016). It starts with a model without any variables, and then sequentially adds variables to the model based on their statistical significance and improvements to the model’s fit. The corrected Akaike Information Criterion (**AICC**) was used as the criterion to choose models in the sequential process, and a significance level of 0.01 was used for variables to be included in the model. A significance level of 0.01 was used in the current study to reduce the chance of type I errors. Sparse variables, which refer to the presence of and include a large number of zeros (not missing values) (Li, 2019), were manually checked and removed after applying the forward selection method.
2. PCA refers to a statistical technique that linearly transforms the original (X) variables into principal components (C) that are uncorrelated vectors derived from the original data (Pearson, 1901). This method can reduce the number of variables and can explain most of the total variation in the original data while removing collinearity. The PROC FACTOR procedure in SAS was used to implement PCA on the numeric variables in the current dataset. A VARIMAX rotation was used to improve factor interpretation, which is an orthogonal rotation method for a more interpretable and simpler structure of the components by maximizing the sum of squares of the factor loadings (Correddu et al., 2021). The number of principal components selected was determined based on the criterion that they cumulatively account for at least 80% of the total variance (Dunteman, 1989).
3. PLS aims to analyze the effects of linear combinations of predictors on a response variable, using latent factors extracted from the predictors to maximize both the explained variance in the response variable and the covariance between the predictors and the response variable (Wold et al., 2001). Compared with PCA, PLS is more beneficial for prediction models as the response variable is included in the process of latent factor extraction (Rougoor et al., 2000). The PROC PLS procedure in SAS was used to implement PLS. To select the optimal number of extracted factors, a randomization-based model comparison test was conducted using the predicted residual sum of squares (PRESS, Font-i-Furnols et al., 2013). A significance level of 0.01 was used.

### Model construction

Based on the variables (forward selection) and factors (PCA, PLS) identified in the previous step, the PROC MIXED procedure of SAS was used to fit linear regression models to predict PDI. Linear regression models aimed to describe the linear relationship between the response variable (*Y*) and several predictors (*X*) as follows:


$$\begin{array}{l} Y=\;\;\;\varepsilon \;+a_{1}X_{1}+\;a_{2}X_{2}\;+\;a_{3}X_{3}\ldots a_{n}X_{n}+e \end{array}$$ (3)

where *Y* represents the transformed response variable (tPDI);  ε  represents the intercept; $X_{1}$, $X_{2}$, $X_{3}$ … $X_{n}$ represent the predictors; and $a_{1}$, $a_{2}$, $a_{3}$ … $a_{n}$ represent the coefficients of the corresponding predictors; and *e* represents the residual error. Diagnostic plots were used to verify the model assumptions, including normally distributed and homogeneously distributed residuals. AICC, *R*-squared, and adjusted *R*-squared scores were calculated as statistical measures to evaluate model fit during building. Compared to *R*-squared, adjusted *R*-squared accounts for model complexity by considering the number of predictors, providing a more balanced measure of model performance. Both *R*-squared and adjusted *R*-squared were used to compare the predicted values with the actual values for the original response variable (PDI) and the transformed response variable (tPDI).

A *k*-fold cross-validation method (Shine et al., 2018) with *k* = 5 was used to fit and evaluate the performance of the above-described model. Data were split into *k* subsets of approximately equal size. The model was then trained and evaluated k times, with each iteration using a different subset as the test set and the remaining *k* − 1 subsets as the training set. Overall model performance was averaged across all *k* iterations, which provides an unbiased estimate of the model’s performance. Additionally, the testing set, which was never used in the model-building process, was used to evaluate the predictive performance of the final models.

### Model evaluation

Measures used to evaluate model performance included the mean absolute error (**MAE**), the mean square prediction error (**MSPE**), and the concordance correlation coefficient (**CCC**).

The MAE is defined as follows:


$$\begin{array}{l} MAE=\;\;\;\frac{1}{n}\underset{i=1}{\overset{n}{\sum }}\;\left|y_{i}-\hat{y_{i}}\right| \end{array}$$ (4)

where *n* represents the number of samples, $y_{i}$ represents the actual value of the *i*th sample, and $\hat{y_{i}}$ represents the predicted value of the *i*th sample.

The MSPE (Bibby and Toutenburg, 1977) has the following equation:


$$\begin{array}{l} MSPE=\;\;\;\frac{1}{n}\underset{i=1}{\overset{n}{\sum }}\;{\left(y_{i}-\hat{y_{i}}\right)}^{2} \end{array}$$ (5)

where *n* represents the number of samples, $y_{i}$ represents the actual value of the *i*th sample and $\hat{y_{i}}$ represents the predicted value of the *i*th sample. RMSPE was used in the current study, which is the square root of MSPE, and puts the error on the data scale. The MSPE was decomposed into ECT (error due to overall bias), ER (error due to the regression slope deviation), and ED (error due to random error). ECT, ER, and ED were calculated using the following equations:


$$\begin{array}{l} ECT=(\bar{P}-\bar{O}) \end{array}$$ (6)

$$\begin{array}{l} ER={\left(S_{p}-R\times S_{o}\right)}^{2} \end{array}$$ (7)

$$\begin{array}{l} ED=(1-R^{2})\times S_{o}^{2} \end{array}$$ (8)

where $\bar{P}$ and $\bar{O}$ are the predicted and observed means, *S*_p_ is the predicted standard deviation, *S*_o_ is the observed standard deviation and *R* is the Pearson product-moment correlation coefficient. Ideally, all error is random error (ED fraction), which indicates the absence of systematic bias or slope deviation in predictions made by the model.

CCC (Lin, 1989) is defined below


$$\begin{array}{l} CCC=R\;\;\;\times C_{b} \end{array}$$ (9)

where *R* is the Pearson correlation coefficient and *C*_b_ is a bias correction factor. The *C*_b_ is calculated as


$$\begin{array}{l} C_{b}\;\;\;=\;\;\;\frac{2}{\left[v\;\;\;+\;\;\;1/v\;\;\;+\;\;\;u^{2}\right]} \end{array}$$ (10)

$$\begin{array}{l} v=\frac{S_{o}}{S_{p}} \end{array}$$ (11)

$$\begin{array}{l} u=\frac{\bar{O\;\;\;}-\bar{P}}{\sqrt{S_{o}\times S_{p}}} \end{array}$$ (12)

where v provides a measure of scale shift, and u provides a measure of location shift.

All measures above were calculated to compare the observed response variable (PDI) against the back-transformed (Box–Cox) predicted tPDI values.

Due to the complexity of the three models, a behavior analysis was conducted to assess how each predictor impacted the predicted back-transformed outcome (Guo et al., 2008). For each model, the mean of each individual predictor (based on training data) was adjusted from −20% to 20% in 5% intervals to identify the direction and magnitude of changes in the response variable (PDI). The change is calculated based on the following equation:


$$\begin{array}{l} p=\frac{A-B\;}{B}\;\times 100\% \end{array}$$ (13)

where *p* is the percentage of PDI change, *A* is the PDI when the predictor changes, and *B* is the PDI without any change of the predictor. This approach was applied to aid in interpretation of predictor impacts, not only due to the application of a data transformation to PDI (tPDI), but also due to the use and application of dimensionality reduction methods (PCA, PLS) which make interpretation of the original variable impacts more challenging.

## Results

### Data processing before variable selection and dimensionality reduction

The optimal *λ* parameter determined for the Box–Cox transformation of the PDI variable estimated by the PROC TRANSREG procedure in SAS was 5.18, which was then used to transform the response variable (PDI) into a more normally distributed response variable (tPDI). Figure S2 shows the distributions of PDI and tPDI.

In terms of outlier removal, two observations were removed because their Cook’s Distance values were greater than 0.5, resulting in 2,147 observations left in the training data for model building.

Four variables, including *Feeder Speed (%)* (*P* = 0.274), *Conditioning Temperature (°C)* (*P* = 0.011), *Expander Power (kWh)* (*P* = 0.051), and *Cooling Time (min)* (*P* = 0.246) were not significantly (*P* > 0.01) related to tPDI at the univariable level and thus removed from further consideration, leaving 51 variables from the original dataset (*n* = 55 variables) for exploration in subsequent steps.

### Variable selection and dimensionality reduction

**Figure 2.**
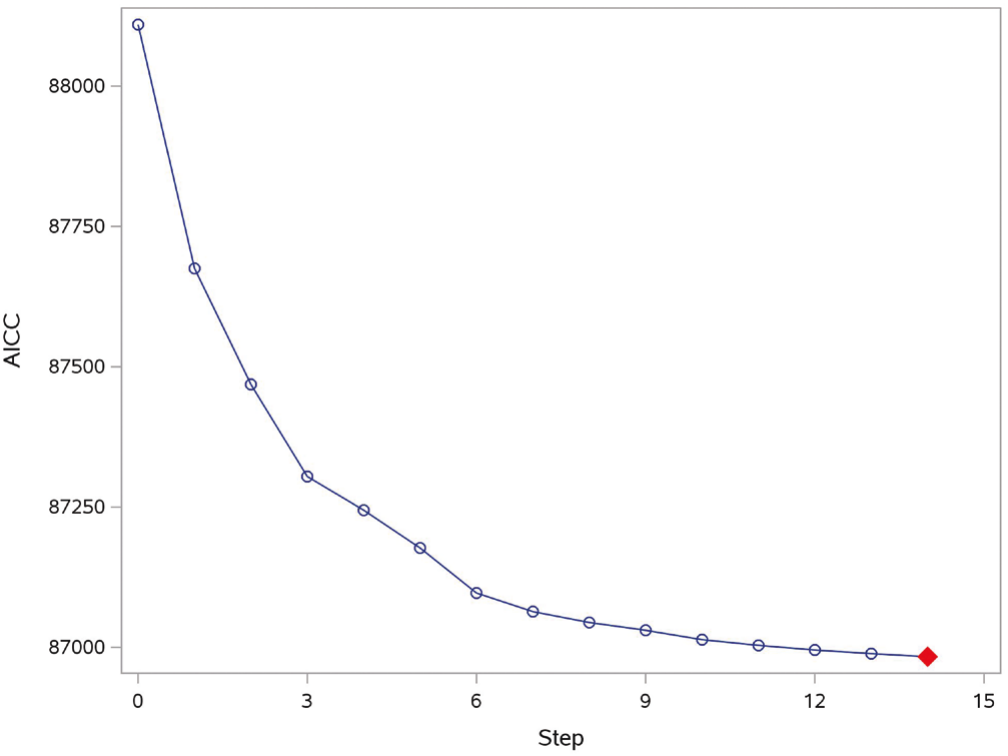
Progression of a forward selection method to choose variables for Model 1. Blue circles represent steps and red diamond represents the step with the lowest AICC.

**Figure 3.**
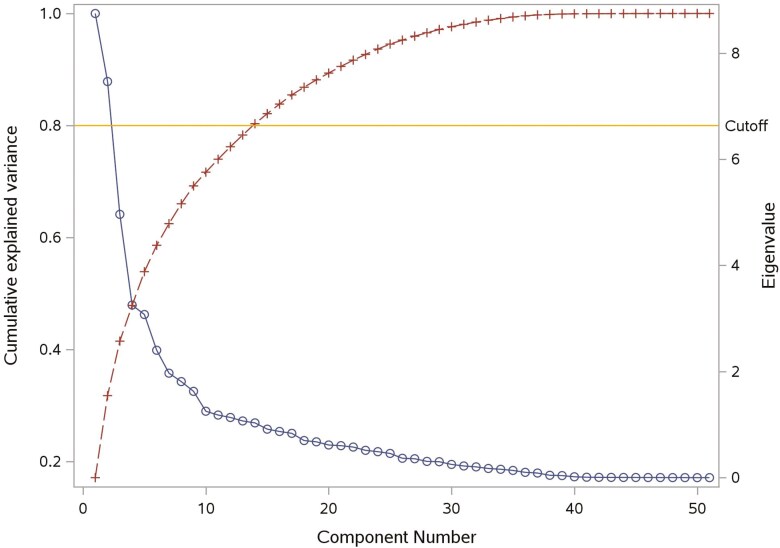
Cumulative explained variance and eigenvalue of components by Principal Component Analysis for Model 2. Brown crosses and line represent the cumulative variance; Blue circles and line represent the eigenvalue.

**Figure 4.**
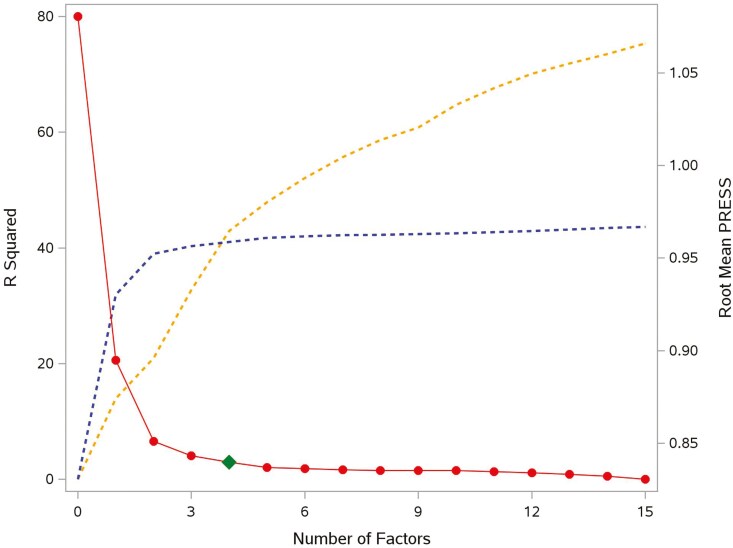
Different numbers of extracted factors of Partial Least Squares using 5-fold cross-validation for Model 3. The red line and points represent the Root Mean PRESS (Predicted Residual Sum of Squares); the green point represents the optimal number of extracted factors; the blue dashed line represents the *R*-squared values of the transformed response variable; the orange dashed line represents the *R*-squared values of the model.

Three variable selection and dimensionality reduction approaches were applied: 1) where 14 variables were selected using the Forward Selection method (Figure 2), and 9 of them were retained as predictors for model construction, excluding 5 sparse variables (*Pellet Binder (%)*, *Soybean Oil (%)*, *Liquid Fat (%)*, *Oat Hulls (%)*, and *Coarse Ground Corn (%)*); 2) where for PCA, the first 14 factors were chosen because their cumulative variance explained exceeded 80% of the total data variance (Figure 3), and 3) where for PLS, 4 factors were extracted from the original data as they achieved the lowest PRESS, and adding additional components did not significantly reduce the PRESS and improve the *R*-squared of the transformed response variable (tPDI) (Figure 4).

**Figure 5.**
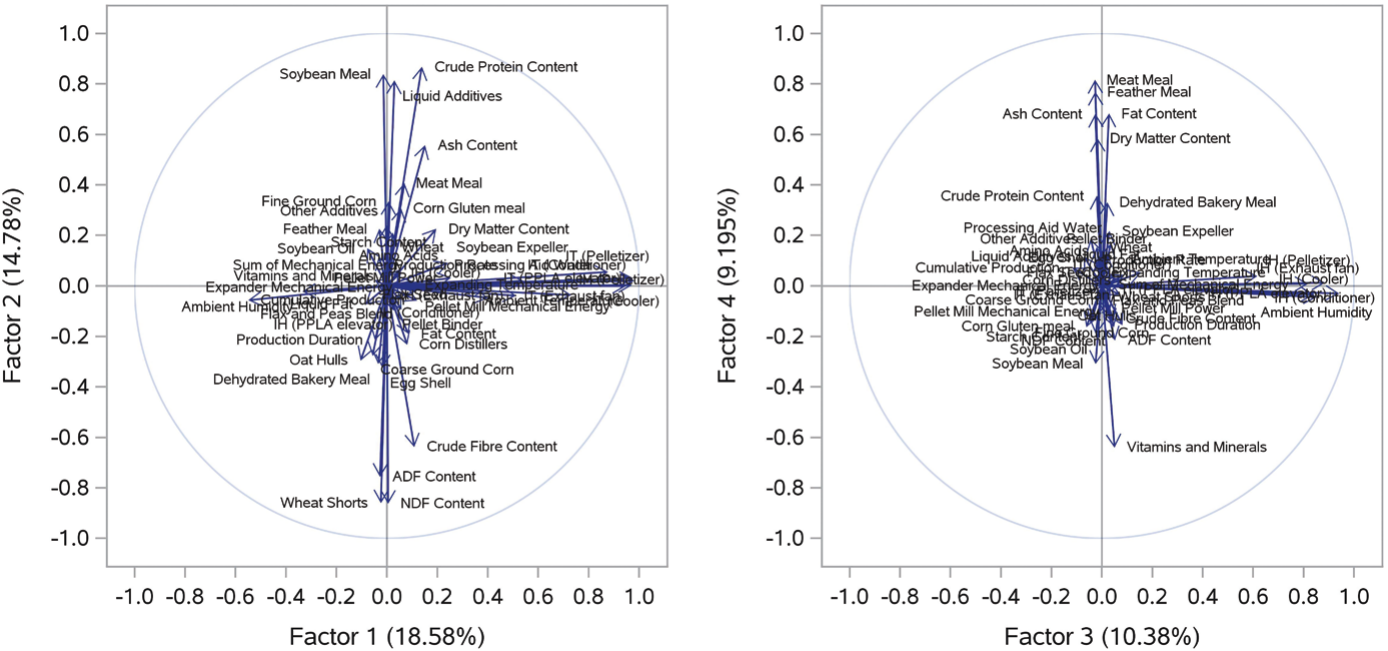
An example of rotated factor loadings from the Principal Component Analysis for Factor 1 vs. Factor 2 (left panel) and Factor 3 vs. Factor 4 (right panel). IH: Indoor Humidity; IT: Indoor Temperature.

**Figure 6.**
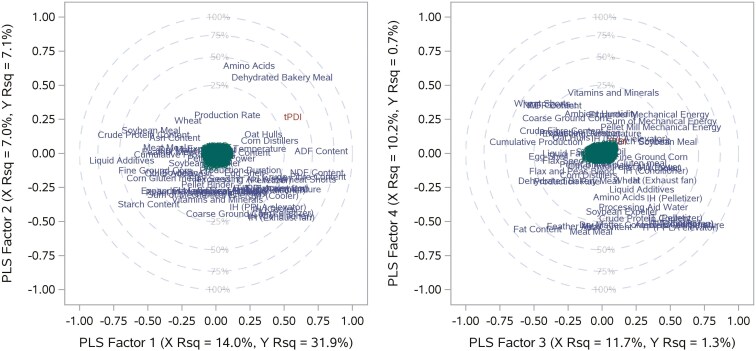
An example of a correlation loading plot from Partial Least Squares for Factor 1 vs. Factor 2 (left panel) and Factor 3 vs. Factor 4 (right panel). IH: Indoor Humidity; IT: Indoor Temperature.

Since PCA and PLS reduced dimensionality (down from *N* = 51 variables) by linearly transforming variables, Figures 5 and 6 are examples of factor loading plots for PCA and PLS, respectively, illustrating the correlation between the factors and the variables.

For PCA analysis, in Figure 5, Factor 1 and Factor 2 explained 18.58% and 14.78% of the total variance, respectively, and Factor 3 and Factor 4 explained 10.38% and 9.195% of the total variance, respectively. Most predictors regarding the EF were positively correlated and positively loaded on Factor 1, like *Indoor Humidity (Pelletizer) (%)* and *Indoor Temperature (Conditioner) (°C)*, and those variables representing FI (e.g., *Soybean Meal (%)*) and NCs (e.g., *NDFContent (%)*) showed either positive or negative loadings on Factor 2.

For PLS analysis, in Figure 6, Factor 1 (31.9%) and Factor 2 (7.0%) accounted for more variance in the transformed response variable (tPDI) compared with Factor 3 (1.3%) and Factor 4 (0.7%). Meanwhile, Factor 1 explained 14.0% of the variance in the predictors, higher than other factors, and most variables had a positive or negative correlation with factor 1. Most predictors were either positively (e.g., *ADF Content (%)*) or negatively (e.g., *Liquid Additives (%)*) loaded on factor 1, while *Amino Acids (%)* and *Dehydrated Bakery Meal (%)* were positively loaded on Factor 2.

### Model fitting

Models to predict tPDI from the variables/factors identified or extracted above via forward selection, PCA and PLS all satisfied the assumptions of normally distributed residuals and constant variance across the response variable, according to the diagnostic plots (Figures S3, S4, and S5). Table 1 shows the fit statistics across the 3 developed models. Model 2 had the best fit among the 3 models, as evidenced by its lowest AICC of 90523.1. Model 3 accounted for a greater amount of variation compared to the other models, according to the adjusted *R*-squared values: for the transformed response variable (tPDI) and its corresponding predicted values, the adjusted *R*-squared value was 0.409; for the original response variable (PDI) and its corresponding back-transformed predicted values, the adjusted *R*-squared score was 0.394. Tables 2, 3, and 4 show the estimated parameters for the 3 models, providing the slopes of the regression coefficients based on the transformed response variable (tPDI). All predictors listed were significant (*P* < 0.01) across the 3 models. In Model 1, *Amino Acids (%)* and *ADF Content (%)* had greater coefficients than other predictors, while *Processing Aid Water (%)* and *Fat Content (%)* had the lowest coefficients. There was no multicollinearity among predictors in model 1, based on VIF values. In Model 2, the coefficients of Factor 1 and Factor 7 were greater than those of other components, and the coefficient of Factor 2 was the lowest. In model 3, the coefficient of Factor 1 was greater than those of other components.

**Table 1.** Comparison of fit statistics of 3 models built (and evaluated) on the training data (80% of the original dataset)

Fit statistics	Model 1^1^	Model 2^2^	Model 3^3^
Number of Predictors	9	14	4
AICC	90,649.4	90,523.1	90,758.9
*R*-squared (tPDI)^4^	0.399	0.380	0.410
Adjusted *R*-squared (tPDI)^4^	0.396	0.375	0.409
*R*-squared (PDI)^5^	0.384	0.365	0.395
Adjusted *R*-squared (PDI)^5^	0.381	0.361	0.394

AICC: corrected Akaike Information Criterion; PDI: Pellet Durability Index; tPDI: transformed Pellet Durability Index.

^1^Model 1 was built based on the subset of variables (*n* = 9) selected by Forward Selection with manual removal of sparse variables.

^2^Model 2 was built based on the subset of predictors (*n* = 14) transformed by Principal Component Analysis.

^3^Model 3 was built based on the subset of predictors (*n* = 4) transformed by Partial Least Squares.

^4^ *R*-squared (tPDI) and adjusted *R*-squared (tPDI) were based on the transformed PDI using Box–Cox method with the corresponding predicted values.

^5^ *R*-squared (PDI) and adjusted *R*-squared (PDI) were based on the original PDI with the corresponding back-transformed predicted values using Box–Cox method.

**Table 2.** Estimated parameters of Model 1^1^ built on the training data (80% of the original dataset)

Effect	Estimate	Standard error	*P*-value	VIF
Intercept	2.14 × 10^9^	1.504 × 10^8^	<0.0001	—
Amino acids (%)	4.42 × 10^8^	7.290 × 10^7^	<0.0001	1.77
ADF content (%)	1.41 × 10^8^	7.623 × 10^6^	<0.0001	1.22
Dehydrated bakery meal (%)	3.06 × 10^7^	2.504 × 10^6^	<0.0001	2.80
Indoor humidity (pelletizer) (%)	1.20 × 10^7^	1.142 × 10^6^	<0.0001	1.84
Expanding temperature (°C)	7.50 × 10^6^	1.276 × 10^6^	<0.0001	1.11
Cumulative production (tonnes)	−5.29 × 10^3^	6.903 × 10^2^	<0.0001	1.25
Ambient humidity (%)	−3.88 × 10^6^	6.995 × 10^5^	<0.0001	1.23
Fat content (%)	−1.55 × 10^8^	1.381 × 10^7^	<0.0001	1.74
Processing aid water (%)	−2.17 × 10^8^	6.158 × 10^7^	0.0004	1.45

VIF: variance inflation factor.

^1^Model 1 was built based on the subset of variables (*n* = 9) selected by Forward Selection with manual removal of sparse variables.

**Table 3.** Estimated parameters of Model 2^1^ built on the training data (80% of the original dataset)

Effect	Estimate	Standard error	*P*-value
Intercept	2.89 × 10^9^	8.43 × 10^6^	<0.0001
Factor 1	1.1 × 10^8^	8.432 × 10^6^	<0.0001
Factor 7	1.08 × 10^8^	8.432 × 10^6^	<0.0001
Factor 8	9.44 × 10^7^	8.432 × 10^6^	<0.0001
Factor 3	4.26 × 10^7^	8.432 × 10^6^	<0.0001
Factor 13	4.17 × 10^7^	8.432 × 10^6^	<0.0001
Factor 12	−4.41 × 10^7^	8.432 × 10^6^	<0.0001
Factor 11	−5.0 × 10^7^	8.432 × 10^6^	<0.0001
Factor 5	−5.52 × 10^7^	8.432 × 10^6^	<0.0001
Factor 4	−5.94 × 10^7^	8.432 × 10^6^	<0.0001
Factor 10	−6.64 × 10^7^	8.432 × 10^6^	<0.0001
Factor 14	−7.07 × 10^7^	8.432 × 10^6^	<0.0001
Factor 6	−7.54 × 10^7^	8.432 × 10^6^	<0.0001
Factor 9	−8.02 × 10^7^	8.432 × 10^6^	<0.0001
Factor 2	−1.55 × 10^8^	8.432 × 10^6^	<0.0001

^1^Model 2 was built based on the subset of predictors (*n* = 14) transformed by Principal Component Analysis.

**Table 4.** Estimated parameters of Model 3^1^ built on the training data (80% of the original dataset)

Effect	Estimate	Standard error	*P*-value
Intercept	2.89 × 10^9^	8.201 × 10^6^	<0.0001
Factor 1	1.05 × 10^8^	3.074 × 10^6^	<0.0001
Factor 2	6.94 × 10^7^	4.329 × 10^6^	<0.0001
Factor 3	2.32 × 10^7^	3.359 × 10^6^	<0.0001
Factor 4	1.86 × 10^7^	3.588 × 10^6^	<0.0001

^1^Model 3 was built based on the subset of predictors (*n* = 4) transformed by Partial Least Squares.

### Model evaluation

**Figure 7.**
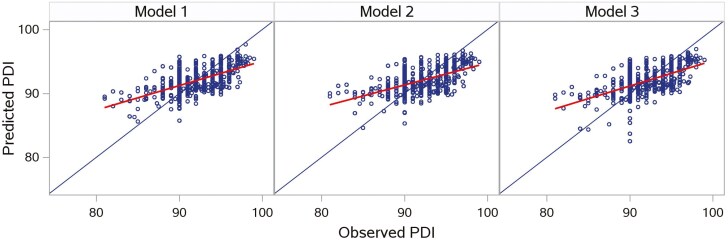
The fit plot of the predicted PDI vs. the observed PDI for all models based on the testing data (20% of the original dataset). The observed PDI refers to the original response variable, and the predicted PDI refers to the back-transformed predicted response variable. The linear relationship for model 1, which was based on the subset of variables (*n* = 9) selected by forward selection with manual removal of sparse variables, is *y* = 0.382*x* + 56.819; for model 2, which was based on the subset of predictors (*n* = 14) transformed by PCA, it is *y* = 0.344*x* + 60.362; for model 3, which was based on the subset of predictors (*n* = 4) transformed by PLS, it is *y* = 0.395*x* + 55.579.

Table 5 shows all 3 model performances based on 5-fold cross-validation using 80% of the original dataset. Overall, Model 3 had a lower MAE (1.90 ± 0.061) and RMSPE (2.42 ± 0.065), as well as the highest CCC (0.565 ± 0.0204), compared with Model 1 and Model 2. The random error (ED%) of all 3 models accounted for the largest fraction of the MSPE. Table 6 shows the model evaluation on the test data representing 20% of the original dataset. Model 1 exhibited better performance than the other two models in terms of MAE (1.96), RMSPE (2.45), and ED (%, 99.9). Model 1 and Model 3 had the same CCC (0.550), higher than Model 2. Model 3 showed slight overfitting due to inconsistent model performance between the training data and testing data. Figure 7 shows all models had a considerable bias of overprediction when the original response variable (PDI) was below 85. While Model 3 had the highest slope (slope = 0.395) in the fit plot, the points predicted by Model 1 were more tightly clustered, indicating a better prediction precision.

**Table 5.** Model performance evaluated by 5-fold cross-validation on the training data (80% of the original dataset)

Measure	Model 1^1^	Model 2^2^	Model 3^3^
MAE	1.93 ± 0.063	1.96 ± 0.081	1.90 ± 0.061
RMSPE	2.45 ± 0.079	2.49 ± 0.087	2.42 ± 0.065
MSPE	5.99 ± 0.389	6.24 ± 0.430	5.84 ± 0.313
ECT (%)	0.6 ± 0.83	0.5 ± 0.76	0.5 ± 0.64
ER (%)	0.1 ± 0.11	0.1 ± 0.12	0.1 ± 0.13
ED (%)	99.3 ± 0.83	99.4 ± 0.71	99.4 ± 0.64
CCC	0.549 ± 0.0273	0.525 ± 0.0229	0.565 ± 0.0204
*C*_b_	0.891 ± 0.0181	0.884 ± 0.0128	0.899 ± 0.0176
*R*	0.616 ± 0.0222	0.594 ± 0.0220	0.628 ± 0.0134

*C*_b_: bias correction factor; CCC: concordance correlation coefficient; ECT (%): the ECT (error due to overall bias) fraction of MSPE; ER (%): the ER (error due to the regression slope deviation) fraction of MSPE; ED (%): the ED (error due to random error) fraction of MSPE; MAE: mean absolute error; MSPE: mean square prediction error; *R*: Pearson correlation coefficient; RMSPE: root mean square prediction error.

^1^Model 1 was built based on the subset of variables (*n* = 9) selected by Forward Selection with manual removal of sparse variables.

^2^Model 2 was built based on the subset of predictors (*n* = 14) transformed by Principal Component Analysis.

^3^Model 3 was built based on the subset of predictors (*n* = 4) transformed by Partial Least Squares.

**Table 6.** Model evaluation on the testing data (20% of the original dataset) that was independent to model construction

Measure	Model 1^1^	Model 2^2^	Model 3^3^
MAE	1.96	2.00	1.97
RMSPE	2.45	2.56	2.49
MSPE	5.99	6.54	6.20
ECT (%)	0.0	0.0	0.0
ER (%)	0.0	0.3	0.7
ED (%)	99.9	99.7	99.3
CCC	0.550	0.501	0.550
*C*_b_	0.899	0.889	0.921
*R*	0.612	0.564	0.597

*C*_b_: bias correction factor; CCC: concordance correlation coefficient; ECT (%): the ECT (error due to overall bias) fraction of MSPE; ER (%): the ER (error due to the regression slope deviation) fraction of MSPE; ED (%): the ED (error due to random error) fraction of MSPE; MAE: mean absolute error; MSPE: mean square prediction error; *R*: Pearson correlation coefficient; RMSPE: root mean square prediction error.

^1^Model 1 was built based on the subset of variables (*n* = 9) selected by Forward Selection with manual removal of sparse variables.

^2^Model 2 was built based on the subset of predictors (*n* = 14) transformed by Principal Component Analysis.

^3^Model 3 was built based on the subset of predictors (*n* = 4) transformed by Partial Least Squares.

Behavior analysis was implemented to reveal the relationship between the change in each predictor and back-transformed PDI while other predictors were held constant. Table 7 shows the behavior analysis of Model 1, indicating that *Expanding Temperature (°C)* had the greatest impact on the PDI: a change of −20%, resulting in a change of −0.943% in PDI, and a change of +20% resulting in a change of 0.907% in PDI. Following *Expanding Temperature (°C)*, *ADF Content (%)*, *Indoor Humidity (Pelletizer) (%)*, and *Dehydrated Bakery Meal (%)* exhibited a positive impact on the PDI while *Fat Content (%)*, *Ambient Humidity (%)*, and *Processing Aid Water (%)* had a negative impact on the PDI. Table 8 shows that Factor 2 had the greatest impact on the PDI in Model 2, being loaded with variables related to NC and FI. In Model 3 (Table 9), Factor 1 had the greatest impact, which was loaded with variables regarding NC and FI.

**Table 7.** Behavior analysis^1^ for Model 1^2^ back transformed to the original response variable (PDI)

Predictor	Predictor change (%)								
	−20	−15	−10	−5	0	5	10	15	20
ADF content (%)	−0.658	−0.492	−0.327	−0.163	0	0.162	0.322	0.482	0.641
Ambient humidity (%)	0.336	0.253	0.169	0.085	0	−0.085	−0.170	−0.255	−0.341
Amino acids (%)	−0.217	−0.163	−0.108	−0.054	0	0.054	0.108	0.162	0.215
Cumulative production (tonnes)	0.145	0.109	0.073	0.036	0	−0.036	−0.073	−0.110	−0.146
Dehydrated bakery meal (%)	−0.250	−0.187	−0.125	−0.062	0	0.062	0.124	0.186	0.248
Expanding temperature (°C)	−0.943	−0.703	−0.467	−0.232	0	0.230	0.458	0.683	0.907
Fat content (%)	0.756	0.570	0.381	0.191	0	−0.193	−0.387	−0.583	−0.781
Indoor humidity (pelletizer) (%)	−0.458	−0.343	−0.228	−0.114	0	0.113	0.226	0.338	0.450
Processing aid water (%)	0.248	0.186	0.124	0.062	0	−0.062	−0.125	−0.187	−0.250

PDI: Pellet Durability Index.

^1^Behavior analysis showed how the percentage change of PDI occurred when a predictor changed. The outcome predicted by the model was tPDI (transformed PDI), which was eventually back-transformed to PDI.

^2^Model 1 was built based on the subset of predictors (*n* = 9) selected by Forward Selection with manual removal of sparse variables.

**Table 8.** Behavior analysis^1^ for Model 2^2^ back transformed to the original response variable (PDI)

Predictor	Predictor change (%)								
	−20	−15	−10	−5	0	5	10	15	20
Factor 1	−0.002	−0.001	−0.001	>−0.001	0	<0.001	0.001	0.001	0.002
Factor 2	−0.003	−0.002	−0.002	−0.001	0	0.001	0.002	0.002	0.003
Factor 3	<0.001	<0.001	<0.001	<0.001	0	>−0.001	>−0.001	>−0.001	>−0.001
Factor 4	0.001	0.001	<0.001	<0.001	0	>−0.001	>−0.001	−0.001	−0.001
Factor 5	0.001	0.001	<0.001	<0.001	0	>−0.001	>−0.001	−0.001	−0.001
Factor 6	<0.001	<0.001	<0.001	<0.001	0	>−0.001	>−0.001	>−0.001	>−0.001
Factor 7	0.001	0.001	0.001	<0.001	0	>−0.001	−0.001	−0.001	−0.001
Factor 8	0.001	0.001	<0.001	<0.001	0	>−0.001	>−0.001	−0.001	−0.001
Factor 9	<0.001	<0.001	<0.001	<0.001	0	>−0.001	>−0.001	>−0.001	>−0.001
Factor 10	0.002	0.001	0.001	<0.001	0	>−0.001	−0.001	−0.001	−0.002
Factor 11	−0.001	−0.001	>−0.001	>−0.001	0	<0.001	<0.001	0.001	0.001
Factor 12	>−0.001	>−0.001	>−0.001	>−0.001	0	<0.001	<0.001	<0.001	<0.001
Factor 13	>−0.001	>−0.001	>−0.001	>−0.001	0	<0.001	<0.001	<0.001	<0.001
Factor 14	0.001	0.001	<0.001	<0.001	0	>−0.001	>−0.001	−0.001	−0.001

PDI: Pellet Durability Index.

^1^Behavior analysis showed how the percentage change of PDI occurred when a predictor changed. The outcome predicted by the model was tPDI (transformed PDI), which was eventually back-transformed to PDI.

^2^Model 2 was built based on the subset of predictors (*n* = 14) transformed by Principal Component Analysis.

**Table 9.** Behavior analysis^1^ for Model 3^2^ back transformed to the original response variable (PDI)

Predictor	Predictor change (%)								
	−20	−15	−10	−5	0	5	10	15	20
Factor 1	−0.002	−0.001	−0.001	>−0.001	0	<0.001	0.001	0.001	0.002
Factor 2	0.001	0.001	0.001	<0.001	0	>−0.001	−0.001	−0.001	−0.001
Factor 3	<0.001	<0.001	<0.001	<0.001	0	>−0.001	>−0.001	>−0.001	>−0.001
Factor 4	>−0.001	>−0.001	>−0.001	>−0.001	0	<0.001	<0.001	<0.001	<0.001

PDI: Pellet Durability Index.

^1^Behavior analysis showed how the percentage change in PDI occurred when a predictor changed. The outcome predicted by the model was tPDI (transformed PDI), which was eventually back-transformed to PDI.

^2^Model 3 was built based on the subset of predictors (*n* = 4) transformed by Partial Least Squares.

## Discussion

### Reflections on model building

Unlike data collected from experiments, the recorded PDI values from the commercial feed mill in the current study exhibited three noteworthy and ‘typical of a commercial operation’ making swine and poultry feed characteristics. Firstly, the distribution of the PDI values was skewed, and there were few observations with PDI values lower than 85 (Figure S2). The skewness could be due to the goal of commercial feed mills, which aim to manufacture pelleted feed with high quality (high PDI values), in comparison to experimental research that may reasonably and intentionally produce pellets of much lower quality (for example, the study reported by Angulo et al. (1996)). Thus, it was necessary to implement a transformation on the response variable (PDI) to address skewness. As a result, whereas one typically could examine the magnitude and direction of slope terms to gauge impact, the nonlinearity introduced via the transformation challenges the interpretation of the estimated parameters of the predictors for model construction. To address this, a behavior analysis was performed that examined % change in the back-transformed output (PDI) relative to % change in inputs, giving a more reasonable understanding of the relative importance and impact of input variables. Secondly, PDI was treated as a continuous numeric variable, but the PDI values for many observations appeared somewhat categorical as they were discrete values like 90, 91, and 92. This could be because some PDI values might have had their decimal parts omitted when measured by operators, which could lead to imprecision in modeling. This explains the presence of bands in the homoscedasticity plots of the 3 models (Figures S3, S4, and S5). Thirdly, the collected data shows that some feed ingredient variables were sparse (Figure S6). This could be due to the complexity of commercial diets where various ingredients can be used routinely in commercial feed mills and some may be used more infrequently. Sparse variables were not incorporated into Model 1 due to the manual checkup and removal after applying the forward selection method. Compared with the forward selection approach, using PCA and PLS could be more effective to manage such sparse variables, by linearly transforming the original predictor variables to new components, and so these variables were retained. Compared with Model 2 and Model 3, Model 1 was more interpretable because it used the selected original variables as predictors for model construction. To reveal how a predictor impacts PDI in Model 1, it is straightforward using the behavior analysis.

### Comparison to extant PDI models

Similar to the 2 previous studies that developed predictive models for PDI (Schroeder et al., 2020; You et al., 2022), the current study considered variables related to MP and FI, as well as EF. However, the current study additionally considered NC of each pelleted batch, more FI as well as internal environmental measurements within the feed mill compared to these previous modeling studies. Like the previous empirical model study by Schroeder et al. (2020), the current study developed linear models for PDI predictions. In contrast, the current study emphasized the criteria for statistical models by considering assumptions, significance, and multicollinearity, and utilized a more robust methodology for model construction and evaluation (*k*-fold cross validation). Compared with the machine learning model study by You et al. (2022), the current study yielded explicit equations with variables and parameters that can be examined and implemented. As such, the current study explored the potential compromise between interpretability and complexity, while simultaneously exploring variable selection and dimensionality reduction methodologies.

While some variables are likely to exhibit nonlinear relationships with tPDI (not explored here), the high dimensionality of the dataset here makes nonlinear modeling complicated. When considering nonlinear relationships (e.g., multiple quadratic terms), or when introducing interactions between variables, it may be more appropriate to use other approaches, especially machine learning, as explored in a previous study (You et al., 2022). From the perspective of model evaluation, the best model (Model 1) in the current study had a CCC of 0.550 whereas the best machine learning model in the study (You et al., 2022) showed a CCC of 0.636. Although there were more variables collected in the current study (*N* = 55) than the machine learning model paper (You et al., 2022) (*N* = 16), machine learning approaches still achieved impressive results. However, the models developed here may represent a simple and easy to implement alternative to such advanced modeling approaches.

In terms of influential variables identified in these extant models compared to the present results, Schroeder et al. (2020) reported amperage, cooler temperature, conditioner temperature, and the inclusion of soybean oil were the most contributed factors to explaining the variance in PDI. In the study by You et al. (2022), average outdoor temperature, bakery byproduct, and wheat inclusion levels were identified as important variables due to their high ranking in feature importance. Based on the behavior analysis for Model 1 developed here, variables found to be important for the prediction of PDI in the current study included *Expanding Temperature (°C)*, *Fat Content (%)*, *ADF Content (%)*, and *Indoor Humidity (Pelletizer) (%)*, among a few other. While studies identified different “specific” variables, commonality can be found across the models, highlighting the importance of fat, manufacturing conditions, environmental conditions, and ingredient selection in the prediction of PDI.

### Interpretation of variables used for PDI prediction

With respect to MP, the current study identified that *Expanding Temperature (°C)*, but also *Cumulative Production (Tonnes*), were significantly related to PDI. The expanding phase of manufacturing pelleted feeds is a highly intensive short-term process with the addition of saturated steam-like conditioning, which can alter the physicochemical properties of feed for further improving the pellet quality (O’Doherty et al., 2001). Implementing expansion prior to pelleting contributes to an increase in PDI, which has been reported by previous studies (Johnston et al., 1999; Lundblad et al., 2009, 2011; Ebbing et al., 2022). However, these studies mainly focused on comparing the impact of expanding vs. not expanding, and there is a lack of research on how expanding temperature influences PDI which was revealed in the current study. *Cumulative Production (Tonnes)* represented the amount of pelleted feed produced since the last die change in the pelletizer, which might indicate the extent of wear of the pellet die. Based on the estimated parameter and behavior analysis, *Cumulative Production (Tonnes)* exhibited a slight negative impact on the PDI, which might intuitively be expected as it may represent “wear” of the pelleting die. To our knowledge, the impact of *Cumulative Production (Tonnes)* on PDI was reported and quantified for the first time here.

Regarding dietary nutrients, *Fat Content (%)* and *ADF Content (%)* were significantly related to PDI. It is well known that the fat level in the diet, including the addition of free liquid oils and solid fats, has significant negative impacts on pellet quality (Briggs et al., 1999; Fahrenholz et al., 2013). Fat or oil blocks the transfer of moisture from the steam treatment into the mash ingredients and prevents gelatinization of starch and denaturation of proteins (Thomas et al., 1998). Cavalcanti and Behnke (2005b) reported the estimated coefficients of fat (including total fat and mixer fat) range between −3.43 and −1.02 in models developed using a response surface methodology, indicating a negative impact on PDI. Similarly, fat also showed a negative impact on PDI in our study. In terms of dietary fiber impacts on PDI, there have been few studies exploring the impact of ADF, though it came up significant here. We postulate that the ADF consisting of insoluble content might improve the feed aggregation, which may be beneficial to PDI.

As for the inclusion of specific ingredients, the current study revealed that *Amino Acids (%)* and *Dehydrated Bakery Meal (%*) were significantly related to PDI. Dunmire et al. (2021) reported that increasing crystalline amino acids improved pellet quality, in agreement with the findings about amino acids in the current study. Bakery meal refers to a mixture of waste from bakery products, such as pasta, potato chip waste, cakes, and breakfast cereals, with NC varying across different regions (Almeida et al., 2011). The current study reported *Dehydrated Bakery Meal (%)* was helpful to improve PDI, which was consistent with the findings from a previous study (Catalá-Gregori et al., 2009) exploring the impact of bakery on pellet quality. *Processing Aid Water (%)* impaired pellet quality in the current study, in contrast to previous studies (Moritz et al., 2003; Hott et al., 2008; Buchanan and Moritz, 2009) that reported adding water could improve PDI by contributing to starch gelatinization. However, other studies (Skoch et al., 1981; Cutlip et al., 2008) reported that water may serve as a lubricant, decreasing friction between the pellet die holes and the roller and thereby decreasing PDI at high levels. It is likely that there may be a nonlinear relationship between PDI and processing aid water not captured by the linear nature of the current analysis.

In terms of the environment, the current study found that *Indoor Humidity (Pelletizer)* and *Ambient Humidity (%)* were significantly related to PDI. The impact of EF on PDI has seldom been reported in previous studies, aside from You et al. (2022) though anecdotally, mill workers reflect upon different challenges pelleting in the winter compared to summer, most likely related to the temperature of raw materials and the temperature/humidity of the air being pulled into the mill. Notably, *Indoor Humidity (Pelletizer)* and *Ambient Humidity (%*) had opposite impacts on the PDI in Model 1. The EF have seldom been reported in previous studies. *Indoor Humidity (Pelletizer) (%)*, capturing the humidity level around the pelletizer, might reflect the environmental conditions influenced by the pelletizer’s operations. During the thermal-mechanical processing, a small amount of steam is typically emitted by the pelletizer and the recorded humidity level was likely associated with the emitted steam. *Ambient Humidity (%*) might be associated with the humidity of cooling air in the cooling process which affects the pellet quality.

### Study limitations

While the current study explored using statistical models to predict pellet quality, there are some limitations to consider. Firstly, only the linear effect of variables was considered in the current study, and it might be helpful to add nonlinear effects and interactions into the modeling. This has been explored previously by this group using Machine Learning (You et al., 2022), which may be a superior approach to addressing complex nonlinearities. Secondly, although an independent subset was used to evaluate the models in the current study, the data was from the same mill and further studies could examine the generalization ability of the models to predict outcomes from other feed mills. Thirdly, certain variables for FI (% inclusion) are naturally sparse, and if not removed can have an impact on parameter estimation in general linear regression models. We applied variable selection (forward selection with manual removal of sparse variables) and dimensionality reduction (PCA and PLS) approaches in the current study, which could reduce the impact of sparse variables. Such variables might be better considered when grouped together, or explored by other modeling methods (e.g., machine learning). Fourthly, the fact that the database more or less contained ‘good’ PDI values (>85) means the models developed may be less practiced under conditions resulting in poor pellet quality (<85). Lastly, while it may initially be difficult to perceive how to implement Model 2 and Model 3 developed herein, the reported loading matrices and estimated parameters can simply be coded into a script (e.g., a Python script), with the output being a prediction of PDI.

## Conclusions

In the current study, a dataset collected from a commercial feed mill was used to construct empirical models to describe the relationship between the PDI and a large number of factors including MP, FI, NC, and EF. The Box–Cox approach was applied to transform the response variable (PDI) to make it more normally distributed for analysis. Forward selection, PCA, and PLS were applied before developing 3 multiple linear regression models. The results indicated that Model 1, built on 9 variables using Forward Selection with manual removal of sparse variables, performed better than the other 2 models. It exhibited consistent model prediction performance on the training data and testing data, in terms of MAE (1.93 ± 0.063 vs. 1.96), RMSPE (2.45 ± 0.079 vs. 2.45), and CCC (0.549 ± 0.0273 vs. 0.550), with a better prediction precision based on the fit plot. A behavior analysis provided a straightforward way to interpret the impact of variables on predicted PDI. In Model 1, *Expanding Temperature (°C)*, *ADF Content (%)*, *Indoor Humidity (Pelletizer) (%)*, and *Dehydrated Bakery Meal (%)* exhibited a positive impact on the PDI while *Fat Content (%)*, *Ambient Humidity (%)*, and *Processing Aid Water (%)* had a negative impact on the PDI. We concluded that Model 1 based on the variables selected by forward selection was the best model to capture the relationship between the PDI and these variables, as well as predict the PDI. The linear analysis in the current study can provide a meaningful comparison against studies that have applied other modeling approaches to address the same problem (e.g., machine learning). The models developed in the current study will help commercial feed mills gain a better understanding of how various variables impact the pellet quality and optimize the manufacturing processes and formulation of pelleted feeds by allowing the prediction of PDI from a combination of formulation, nutrition, manufacturing, and environmental conditions.

## Supplementary Material

skaf021_suppl_Supplementary_Material

## Abbreviations

AICC: corrected Akaike Information Criterion
ANOVA: analysis of variance
CCC: concordance correlation coefficient
MSPE: mean square prediction error
PLS: partial least squares
PCA: principal component analysis
PDI: pellet durability index
tPDI: transformed pellet durability index
RMSPE: root mean square prediction error
