# Supplementary material for: Prediction of Pellet Durability Index in a commercial feed mill using multiple linear regression with variable selection and dimensionality reduction
Source: J Anim Sci. 2025 Feb 4;103:skaf021. doi: 10.1093/jas/skaf021 (PMC11914881; doi:10.1093/jas/skaf021)
Supplement: skaf021_suppl_Supplementary_Material [file skaf021_suppl_supplementary_material.docx]

**Table S1** The description of data collected from the feed mill of Trouw Nutrition Canada.

| Index | Category | Variable | Unit | Mean | SD | Minimum | Median | Maximum |
| --- | --- | --- | --- | --- | --- | --- | --- | --- |
| 1 | EF | Ambient Humidity | % | 65.19 | 13.19 | 22.50 | 66.63 | 91.83 |
| 2 | EF | Ambient Temperature | ℃ | 11.14 | 10.82 | -16.08 | 11.50 | 33.25 |
| 3 | EF | Indoor Humidity (Conditioner) | % | 36.84 | 7.86 | 18.00 | 36.25 | 60.34 |
| 4 | EF | Indoor Humidity (Cooler) | % | 49.38 | 9.21 | 26.08 | 48.83 | 74.75 |
| 5 | EF | Indoor Humidity (Exhaust fan) | % | 24.19 | 6.50 | 10.88 | 23.50 | 47.88 |
| 6 | EF | Indoor Humidity (PPLA elevator) | % | 47.65 | 9.01 | 20.95 | 47.34 | 74.17 |
| 7 | EF | Indoor Humidity (Pelletizer) | % | 28.20 | 9.76 | 10.50 | 27.25 | 55.07 |
| 8 | EF | Indoor Temperature (Conditioner) | ℃ | 21.67 | 8.30 | 1.92 | 21.75 | 37.75 |
| 9 | EF | Indoor Temperature (Cooler) | ℃ | 16.13 | 8.61 | -9.83 | 17.17 | 30.17 |
| 10 | EF | Indoor Temperature (Exhaust fan) | ℃ | 29.10 | 7.79 | 6.90 | 29.17 | 44.25 |
| 11 | EF | Indoor Temperature (PPLA elevator) | ℃ | 17.11 | 9.50 | -7.80 | 17.44 | 36.25 |
| 12 | EF | Indoor Temperature (Pelletizer) | ℃ | 28.63 | 4.98 | 11.33 | 28.25 | 40.84 |
| 13 | FI | Amino Acids | % | 0.37 | 0.15 | 0.00 | 0.36 | 1.04 |
| 14 | FI | Coarse Ground Corn | % | 1.48 | 6.18 | 0.00 | 0.00 | 44.46 |
| 15 | FI | Corn Distillers | % | 1.19 | 3.07 | 0.00 | 0.00 | 18.25 |
| 16 | FI | Corn Gluten meal | % | 0.46 | 0.88 | 0.00 | 0.00 | 7.38 |
| 17 | FI | Dehydrated Bakery Meal | % | 6.08 | 5.53 | 0.00 | 5.89 | 16.45 |
| 18 | FI | Egg Shell | % | 0.57 | 1.31 | 0.00 | 0.00 | 8.75 |
| 19 | FI | Feather Meal | % | 0.74 | 1.12 | 0.00 | 0.00 | 6.34 |
| 20 | FI | Fine Ground Corn | % | 46.08 | 10.94 | 0.00 | 46.76 | 69.53 |
| 21 | FI | Flax Seed | % | 0.07 | 0.84 | 0.00 | 0.00 | 10.81 |
| 22 | FI | Flax and Peas Blend | % | 0.12 | 1.26 | 0.00 | 0.00 | 14.70 |
| 23 | FI | Liquid Additives | % | 0.32 | 0.22 | 0.00 | 0.26 | 0.99 |
| 24 | FI | Liquid Fat^1^ | % | 0.06 | 0.19 | 0.00 | 0.00 | 1.98 |
| 25 | FI | Meat Meal | % | 2.73 | 3.67 | 0.00 | 0.00 | 11.28 |
| 26 | FI | Oat Hulls | % | 0.31 | 1.10 | 0.00 | 0.00 | 9.37 |
| 27 | FI | Other Additives | % | 0.01 | 0.03 | 0.00 | 0.00 | 0.20 |
| 28 | FI | Pellet Binder | % | 0.00 | 0.05 | 0.00 | 0.00 | 0.54 |
| 29 | FI | Processing Aid Water | % | 0.86 | 0.16 | 0.00 | 0.84 | 1.51 |
| 30 | FI | Soybean Expeller | % | 2.93 | 3.47 | 0.00 | 2.48 | 15.14 |
| 31 | FI | Soybean Meal | % | 13.57 | 8.56 | 0.00 | 14.79 | 37.42 |
| 32 | FI | Soybean Oil | % | 0.02 | 0.11 | 0.00 | 0.00 | 2.02 |
| 33 | FI | Vitamins and Minerals | % | 1.49 | 1.68 | 0.09 | 0.91 | 9.74 |
| 34 | FI | Wheat | % | 11.07 | 8.07 | 0.00 | 10.21 | 52.03 |
| 35 | FI | Wheat Shorts | % | 7.99 | 11.43 | 0.00 | 0.12 | 41.95 |
| 36 | MP | Cumulative Production | Tonnes | 20589.33 | 13432.40 | 35.85 | 18579.22 | 47953.85 |
| 37 | MP | Production Duration | Hour | 1.26 | 0.72 | 0.31 | 1.08 | 5.92 |
| 38 | MP | Conditioning Temperature | ℃ | 73.05 | 6.20 | 30.50 | 73.00 | 93.70 |
| 39 | MP | Cooling Time | Minutes | 13.77 | 1.50 | 9.20 | 13.60 | 25.00 |
| 40 | MP | Expander Mechanical Energy | kWh/Tonne | 8.48 | 1.45 | 3.80 | 8.30 | 25.00 |
| 41 | MP | Expander Power | kWh | 178.39 | 20.59 | 76.80 | 181.10 | 227.80 |
| 42 | MP | Expanding Temperature | ℃ | 92.01 | 6.85 | 62.50 | 91.80 | 111.70 |
| 43 | MP | Feeder Speed | % | 54.68 | 5.68 | 23.00 | 55.00 | 91.00 |
| 44 | MP | Pellet Mill Mechanical Energy | kWh/Tonne | 10.31 | 2.41 | 6.10 | 9.90 | 33.00 |
| 45 | MP | Pellet Mill Power | kW | 214.89 | 22.80 | 119.40 | 216.20 | 320.50 |
| 46 | MP | Production Rate | Tonne/hour | 21.65 | 2.99 | 6.00 | 22.00 | 32.50 |
| 47 | MP | Sum of Mechanical Energy | kWh/Tonne | 18.74 | 3.29 | 12.50 | 18.20 | 45.90 |
| 48 | NC | ADF Content | % | 3.45 | 1.20 | 1.29 | 2.95 | 7.25 |
| 49 | NC | Ash Content | % | 5.11 | 1.10 | 2.60 | 4.73 | 8.43 |
| 50 | NC | Crude Fibre Content | % | 2.83 | 1.20 | 1.37 | 2.30 | 9.24 |
| 51 | NC | Crude Protein Content | % | 16.94 | 3.24 | 7.75 | 17.32 | 26.72 |
| 52 | NC | Dry Matter Content | % | 85.21 | 3.27 | 61.62 | 85.79 | 89.27 |
| 53 | NC | Fat Content | % | 3.69 | 0.80 | 2.07 | 3.67 | 7.46 |
| 54 | NC | NDF Content | % | 9.39 | 3.58 | 4.74 | 7.64 | 19.89 |
| 55 | NC | Starch Content | % | 38.27 | 4.85 | 22.77 | 38.40 | 54.63 |
| 56 | - | PDI | % | 91.88 | 3.11 | 80.00 | 92.00 | 99.00 |

EF: Environmental Factor; FI: Feed Ingredient (as fed); IH: Indoor Humidity; IT: Indoor Temperature; MP: Manufacturing Parameter; NC: Nutrient Composition (as fed); PDI: Pellet Durability Index; PPLA: Post-pellet liquid application.

^1^ Liquid Fat refers to the liquid fat added to the mixer.


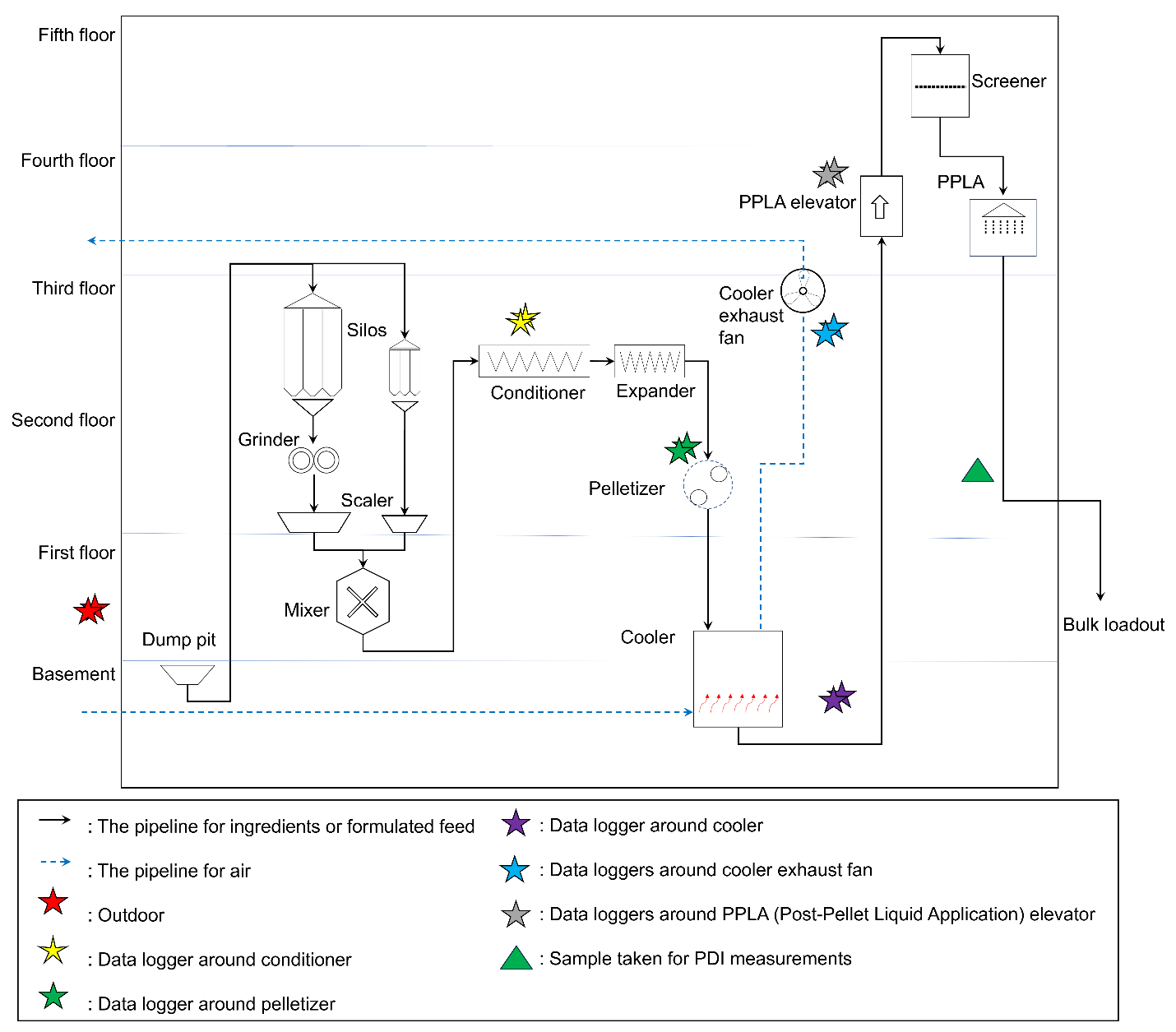


**Figure S1** Feed plant schematic diagram with data logger locations for environmental factor collection.


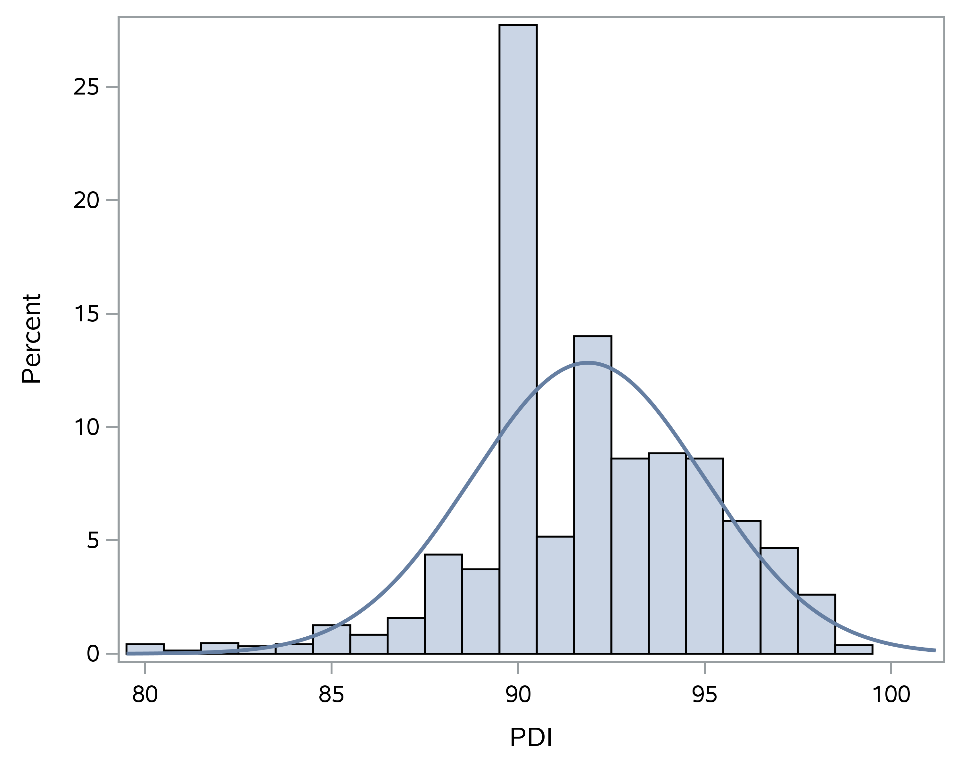

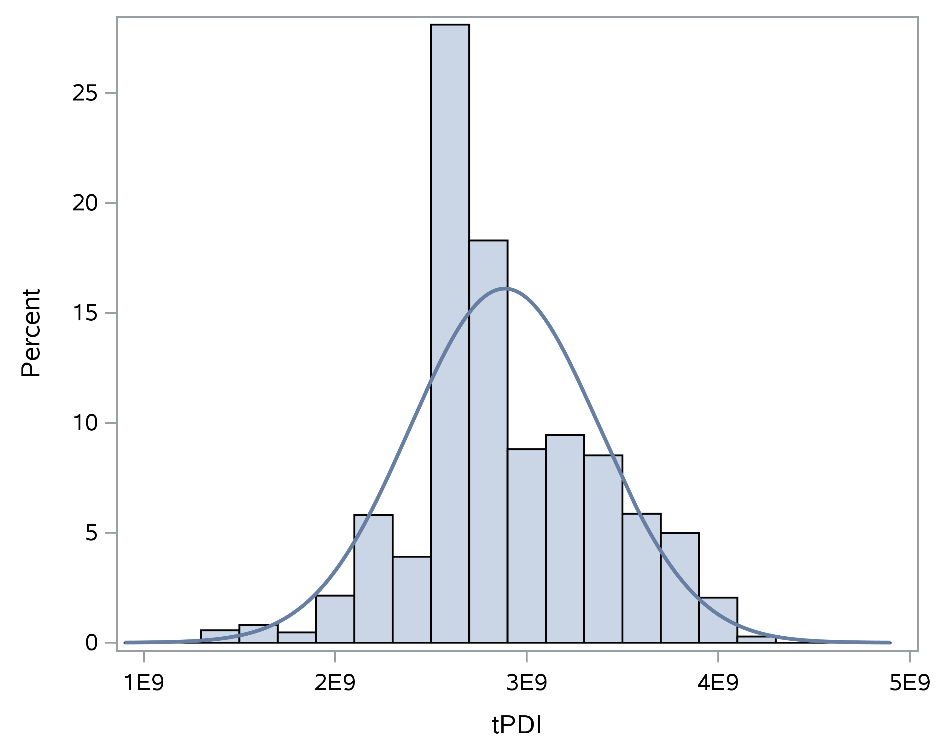


**Figure S2** Distributions of PDI (left panel) and tPDI (right panel).

PDI: Pellet Durability Index; tPDI: transformed Pellet Durability Index by Box-Cox transformation method.


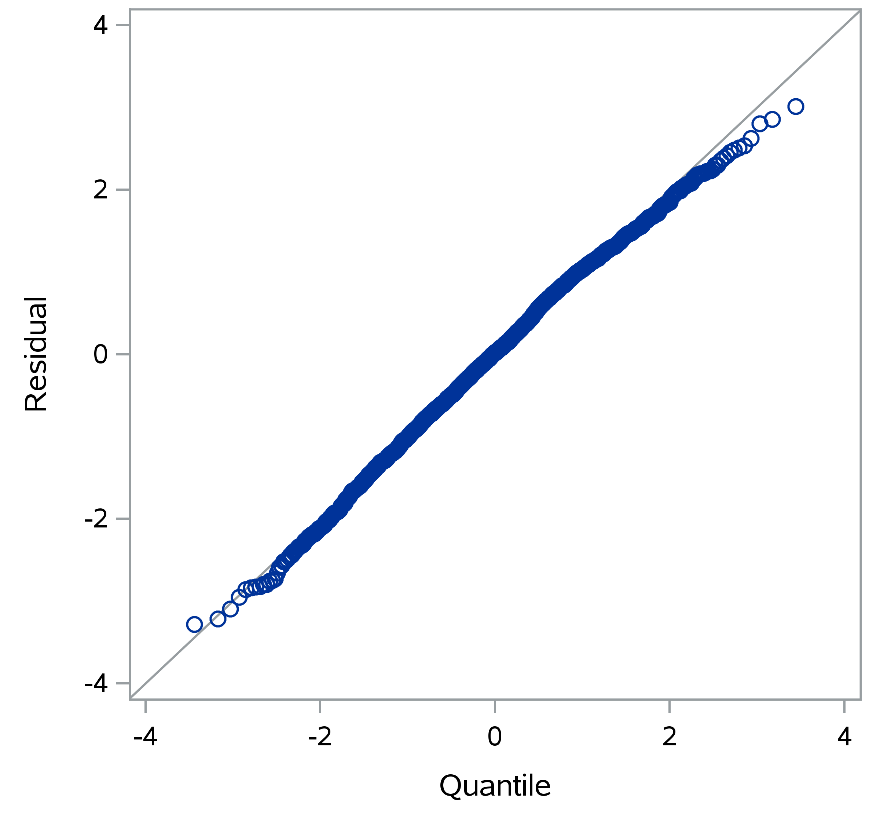

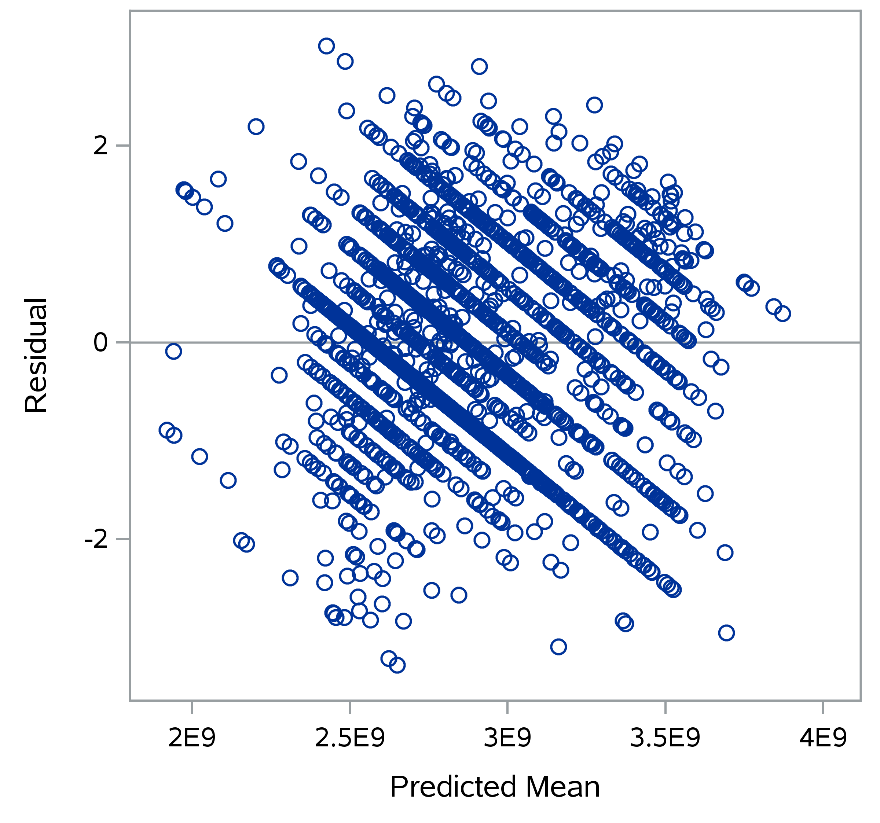


Figure S3 Diagnostic plots for Model 1: Studentized residual Quantile-Quantile plot (left panel) and homoscedasticity plot (right panel).

Model 1 was built based on the subset of variables (n = 14) selected by Forward Selection with manual removal of sparse variables.


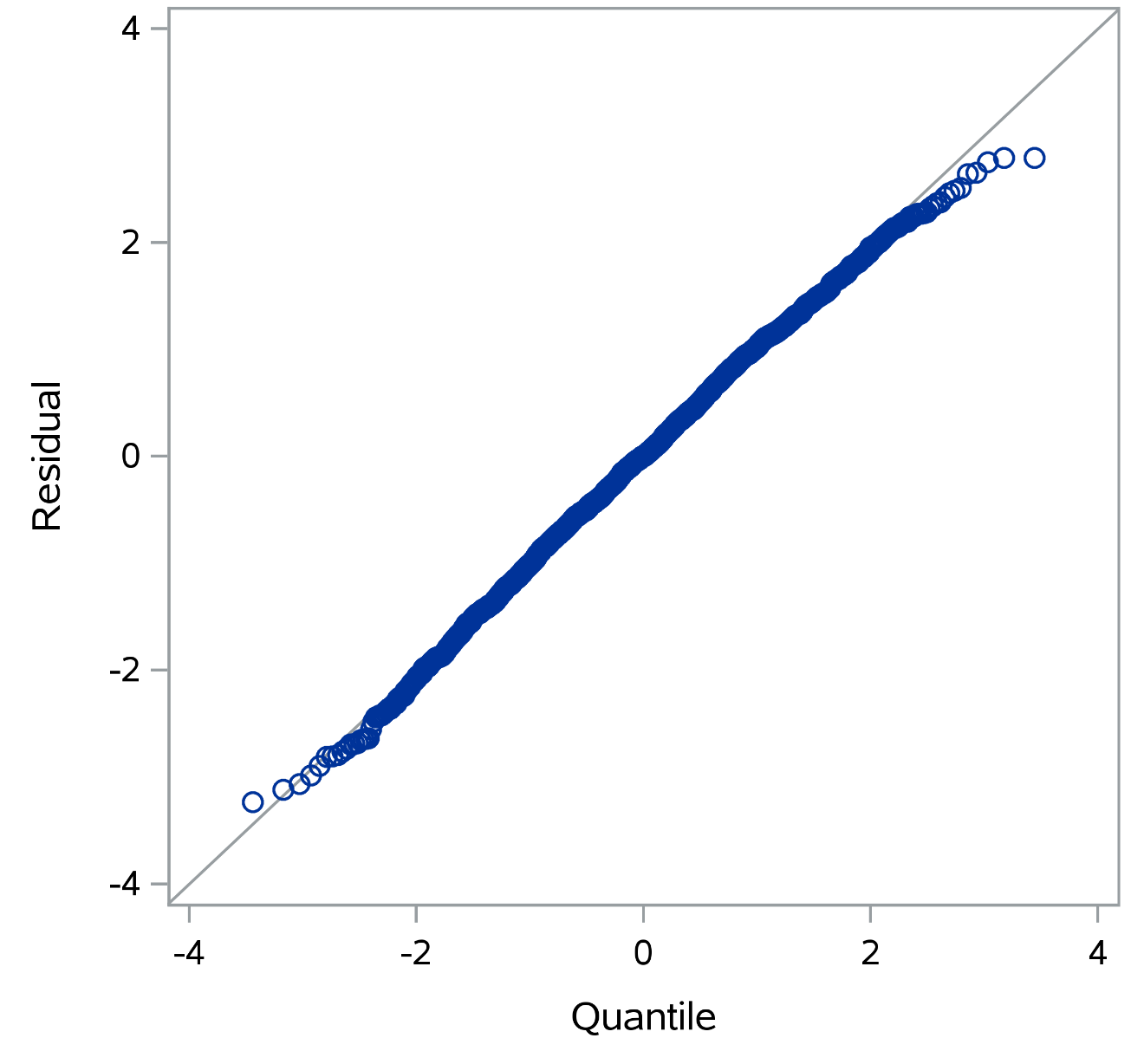

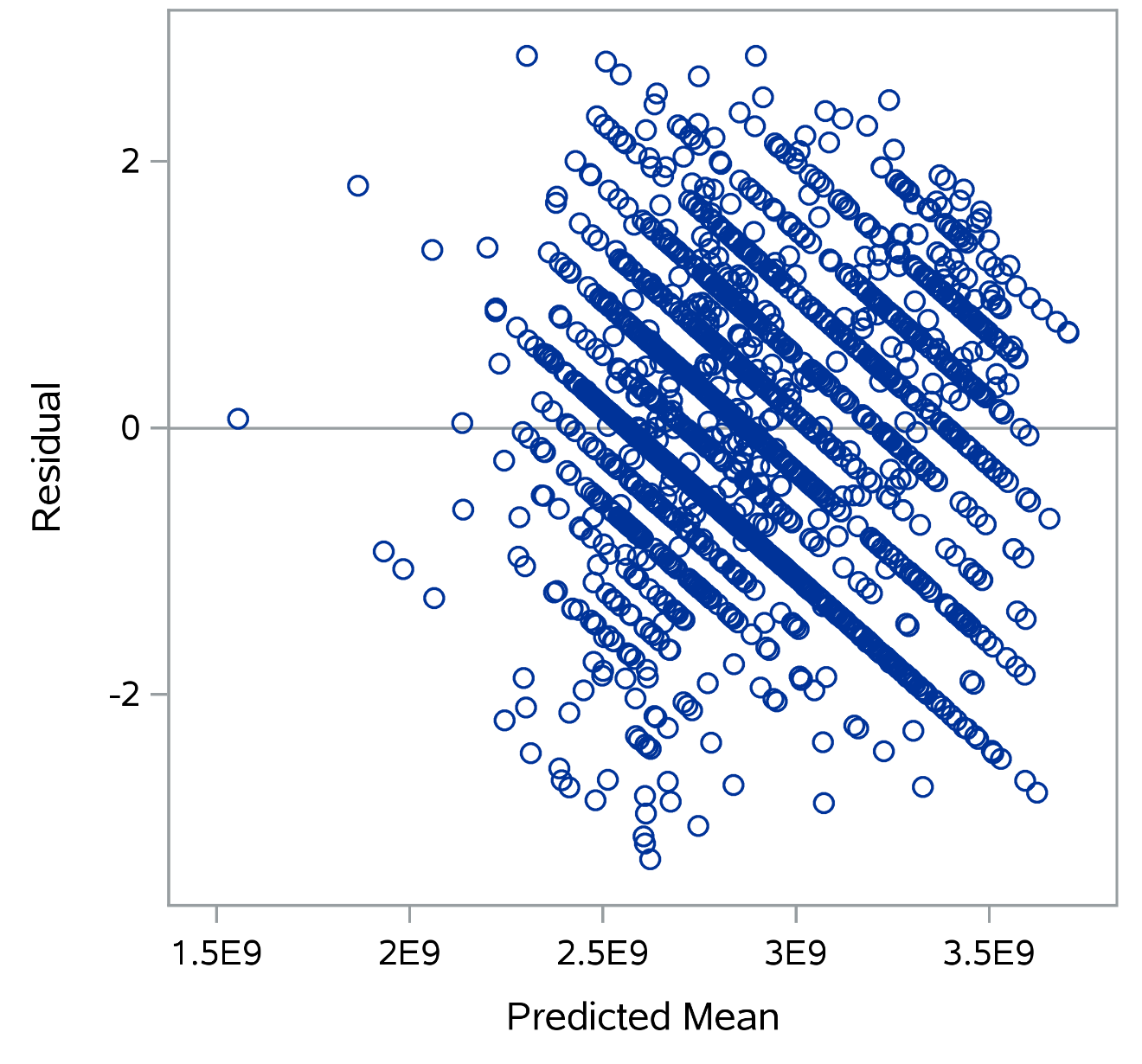


Figure S4 Diagnostic plots for Model 2: Studentized residual Quantile-Quantile plot (left panel) and homoscedasticity plot (right panel).

Model 2 was built based on the subset of factors (n = 14) transformed by Principal Component Analysis.


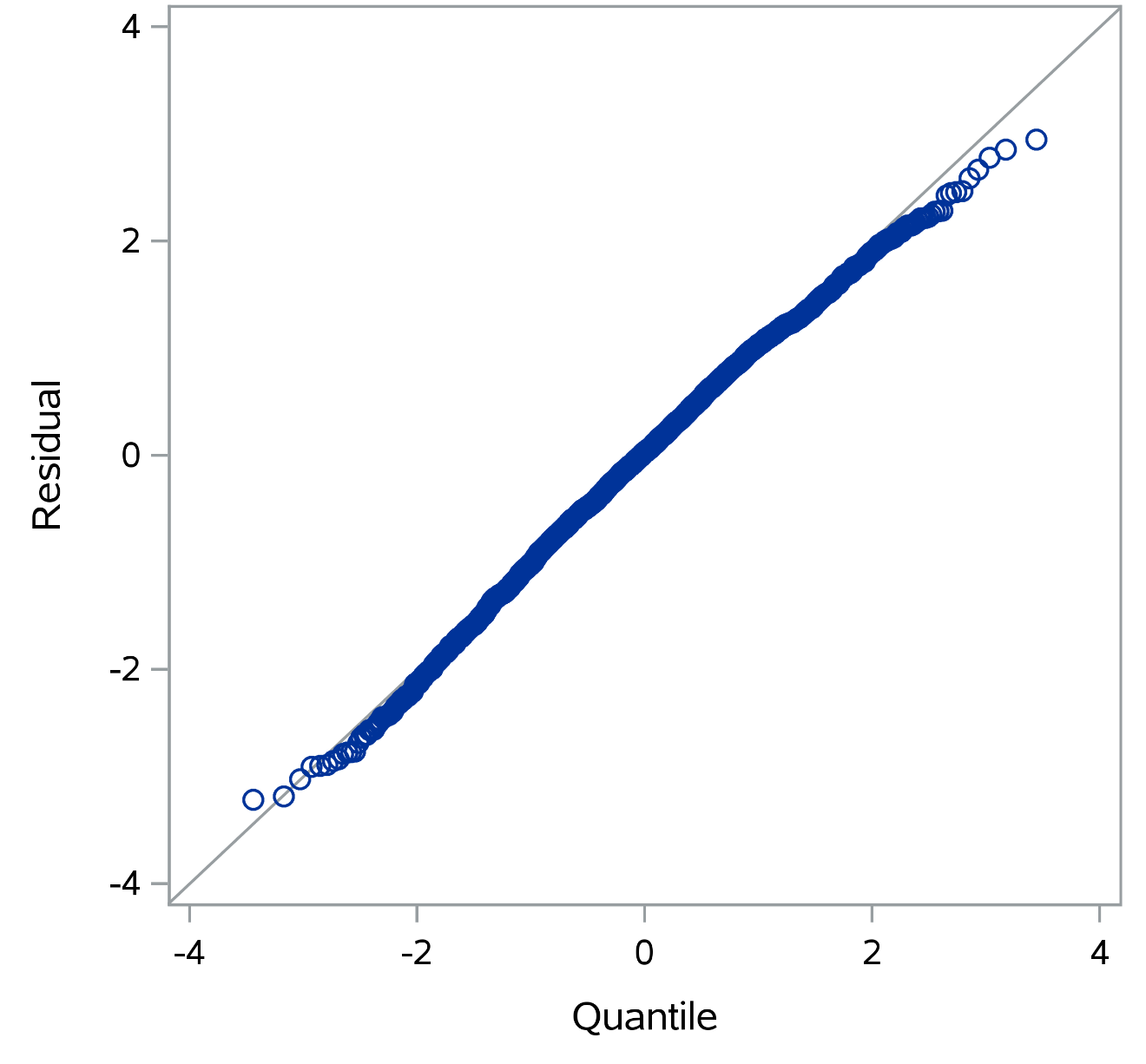

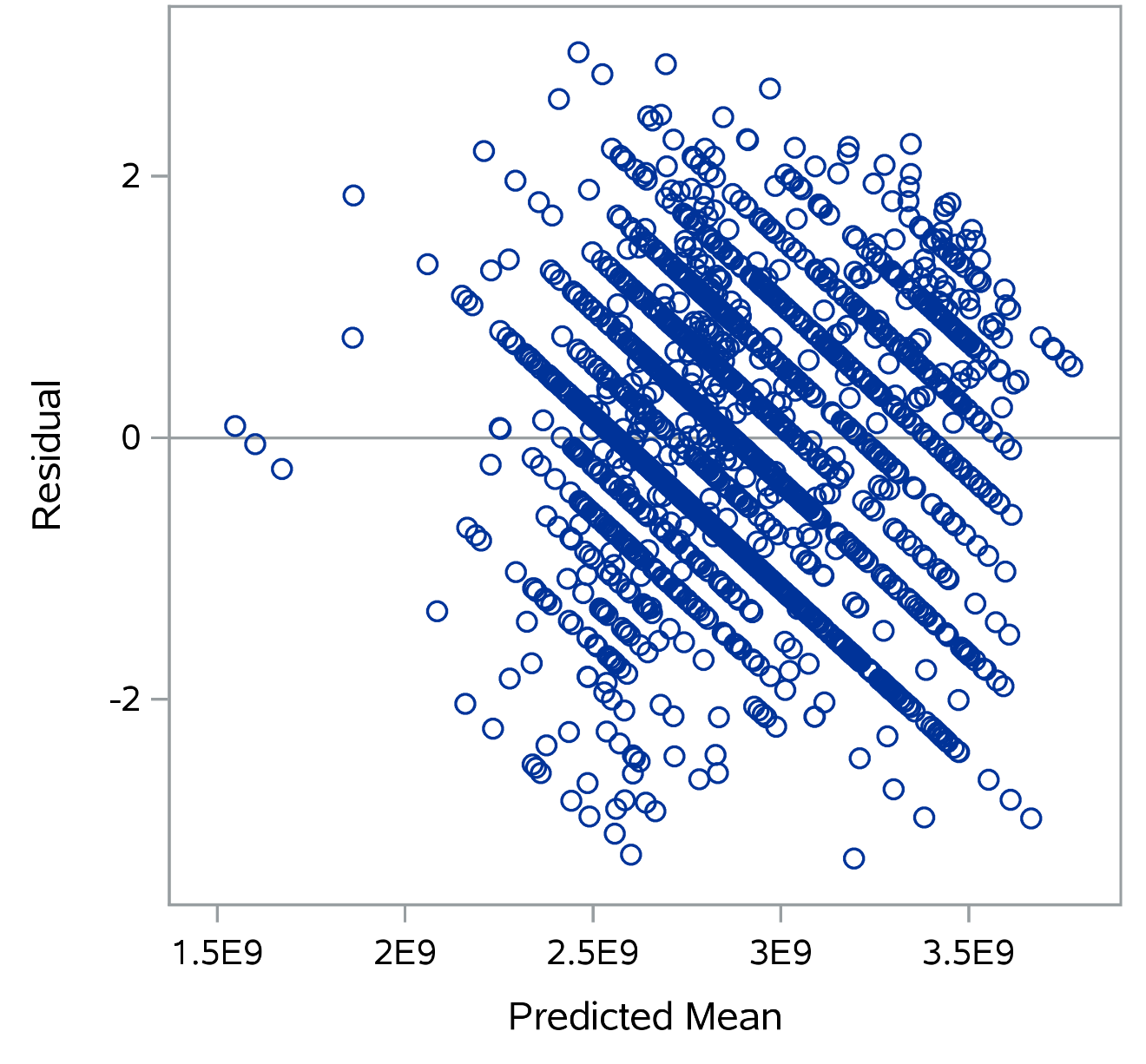


Figure S5 Diagnostic plots for Model 3: Studentized residual Quantile-Quantile plot (left panel) and homoscedasticity plot (right panel).

Model 3 was built based on the subset of factors (n = 4) transformed by Partial Least Squares.


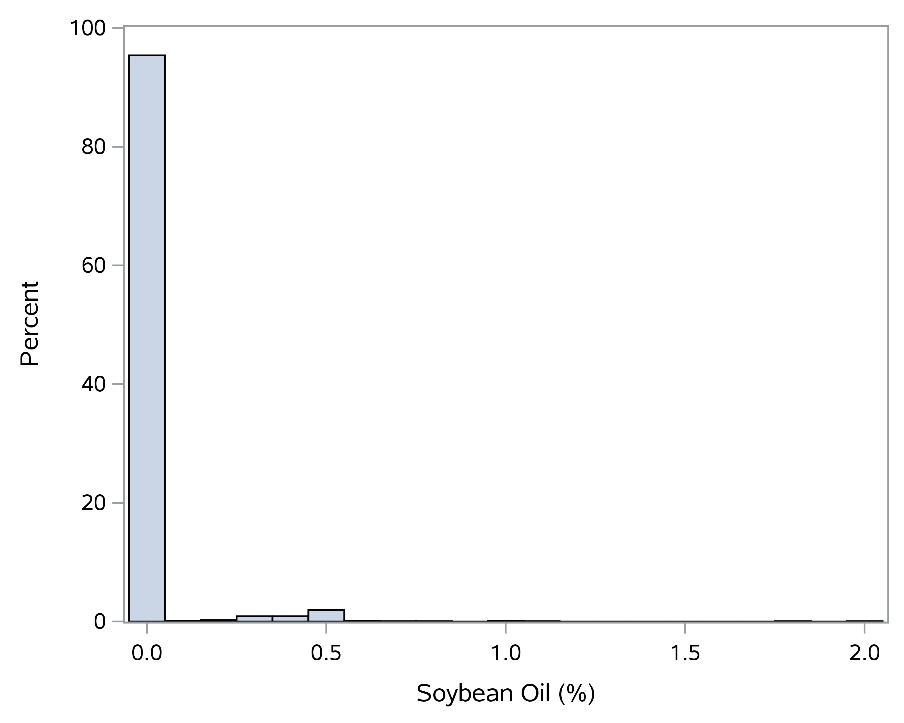

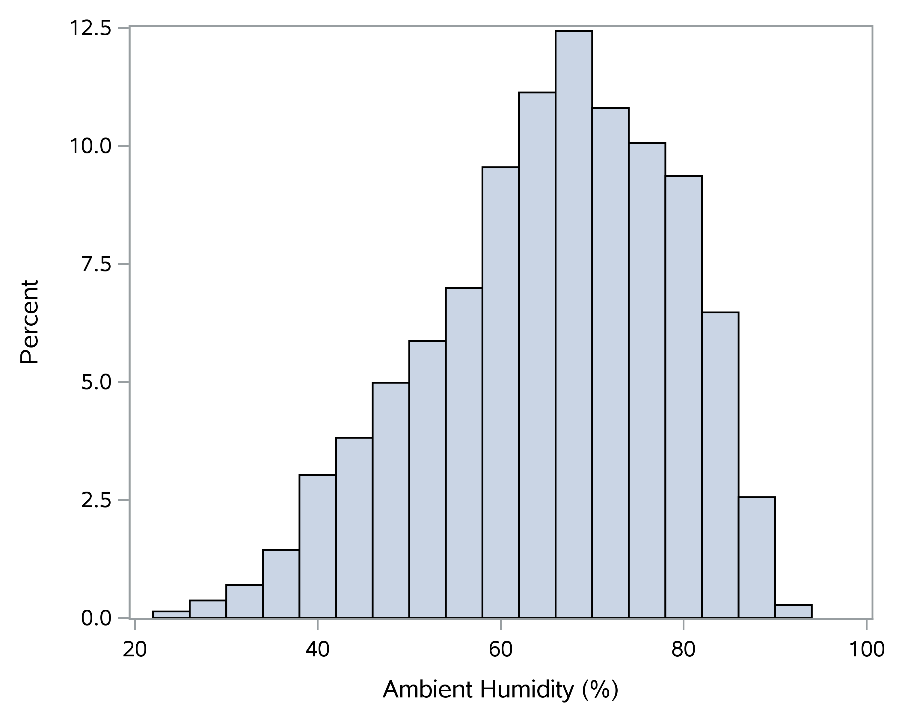


**Figure S6** Distributions of a sparse variable (*Soybean Oil (%),* left panel) and a non-sparse variable (*Ambient Humidity (%),*right panel).
